# Combined RNA/tissue profiling identifies novel Cancer/testis genes

**DOI:** 10.1002/1878-0261.12900

**Published:** 2021-06-23

**Authors:** Soazik P. Jamin, Feria Hikmet, Romain Mathieu, Bernard Jégou, Cecilia Lindskog, Frédéric Chalmel, Michael Primig

**Affiliations:** ^1^ Inserm, EHESP, Irset (Institut de recherche en santé, environnement et travail) – UMR_S, Univ Rennes France; ^2^ Department of Immunology, Genetics and Pathology Science for Life Laboratory Rudbeck Laboratory Uppsala University Sweden; ^3^ Department of Urology University Hospital Rennes France

**Keywords:** Cancer/testis genes, GeneChip, Oncogenes, RNA‐Sequencing, Tissue microarrays

## Abstract

Cancer/Testis (CT) genes are induced in germ cells, repressed in somatic cells, and derepressed in somatic tumors, where these genes can contribute to cancer progression. CT gene identification requires data obtained using standardized protocols and technologies. This is a challenge because data for germ cells, gonads, normal somatic tissues, and a wide range of cancer samples stem from multiple sources and were generated over substantial periods of time. We carried out a GeneChip‐based RNA profiling analysis using our own data for testis and enriched germ cells, data for somatic cancers from the Expression Project for Oncology, and data for normal somatic tissues from the Gene Omnibus Repository. We identified 478 candidate loci that include known CT genes, numerous genes associated with oncogenic processes, and novel candidates that are not referenced in the Cancer/Testis Database (www.cta.lncc.br). We complemented RNA expression data at the protein level for SPESP1, GALNTL5, PDCL2, and C11orf42 using cancer tissue microarrays covering malignant tumors of breast, uterus, thyroid, and kidney, as well as published RNA profiling and immunohistochemical data provided by the Human Protein Atlas (www.proteinatlas.org). We report that combined RNA/tissue profiling identifies novel CT genes that may be of clinical interest as therapeutical targets or biomarkers. Our findings also highlight the challenges of detecting truly germ cell‐specific mRNAs and the proteins they encode in highly heterogenous testicular, somatic, and tumor tissues.

## Graphical abstract

Cancer/Testis (CT) genes are induced in germ cells, repressed in somatic cells, and derepressed in somatic tumors where they can contribute to cancer progression. We report that combined RNA/tissue profiling identifies novel CT genes with potential oncogenic functions and highlight the challenges of detecting germ cell‐specific mRNAs and the proteins they encode in highly heterogenous testicular, somatic, and tumor tissues.

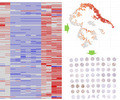

**Abbreviations**
CT genes: Cancer/testis genes
TMAs: Tissue microarrays

## 1 Introduction

Cancer/Testis (CT) genes are expressed in testicular cells and in somatic cancers but typically not in their corresponding normal somatic tissues, reviewed in Ref. [1]. The majority of CT genes function in gametogenesis and fertility, and their abnormal expression in somatic cancer cells can contribute to malignant properties. Indeed, previous work has identified CT genes that are essential for cancer cell division and that affect regulatory signal transduction pathways [2]. Gaining insight into CT gene's biological roles helps better understand how cancer cells proliferate, form metastases, repair DNA damage, suppress apoptosis, alter signaling pathways, and invade normal tissues; for review, see Ref. [3]. More recently, CT genes were proposed to be biomarkers for cancer stem cells that are thought to play roles in the maintenance of tumor growth and resistance to chemotherapy, reviewed in Ref. [4].

Molecular biological and genomic approaches have led to the discovery of several hundred CT genes referenced in the CT database (www.cta.lncc.br) [5]. These loci were initially classified into testis‐specific, testis/brain‐specific, and testis‐selective groups (whereby low expression in two nontesticular tissues is observed); it is noteworthy that the majority of CT genes has been identified via mRNA expression in limited somatic control sample sets, which may explain why they are frequently not genuinely testis‐specific; for a detailed discussion, see Ref. [6]. It is therefore currently unclear how many CT genes are indeed expressed only in male gonads and somatic cancers both at the RNA and protein levels. More recent work carried out by the group developing the Human Protein Atlas, which monitors protein localization in all normal human tissues and numerous cancers, has made a major contribution to determining the human testicular proteome. This study also revealed the somatic cancer expression profile of testicular proteins, some of which are relevant for non‐small‐cell lung cancer [7, 8].

We report a combined microarray‐based RNA/tissue profiling analysis of somatic cancers, normal tissues, prepubertal and adult testis biopsies, total testis samples, and enriched meiotic and postmeiotic germ cells. The approach identified most known and also novel CT genes in addition to oncogenes and cancer‐associated genes that had not been profiled in the male germline before. We selected promising cases and further characterized the proteins they encode using testicular sections and cancer tissue microarrays. Our results represent a rich source for further functional analyses of CT genes in the field of molecular oncology.

## 2 Materials and methods

### 2.1 GeneChip RNA profiling data assembly

The entire dataset was generated with Affymetrix Human Genome U133 Plus 2.0 GeneChip (Thermo Fisher, Courtaboeuf, France). Expression data for human testis (total testis and isolated seminiferous tubules), prepubertal biopsies (high, intermediate, and low infertility risk; HIR, IIR, and LIR), adult testicular biopsies with different Johnson scores indicating the steps where spermatogenesis is disrupted (JS1, JS2, JS3, JS5, JS7, JS8, and JS10) and enriched germ cells (pachytene spermatocytes and round spermatids) were described in reference [9]. Expression data for 45 normal somatic control tissues were downloaded from the NCBI's Gene Omnibus (GEO: GSE7307, GSE6565, and GSE11839) repository [10]; see Supplemental File S1 columns BZ to DR for tissue‐type annotation data. Cancer expression data for 214 cancer subtypes produced by the Expression for Oncology (expO) project (www.intgen.org) were retrieved from GEO (GSE2109) and combined with two other datasets (GSE10802 and GSE6891); see Supplemental File S1 columns DT to LN for cancer types.

### 2.2 GeneChip data processing and analysis

GeneChip U133 Plus 2.0 expression data were quality‐controlled, processed, and normalized as in reference [9, 11]. After quality control, raw data CEL files were normalized, background‐corrected, and summarized with the robust multiarray average function implemented in AMEN [12].

Next, we defined classes of transcripts according to their expression pattern in normal and cancer tissue samples. Transcripts specifically expressed (SE) or preferentially expressed (PE) in a given normal tissue were identified by applying three filtration steps. First, intensity signal is above the background expression cutoff (BEC = 5.5, corresponding to the overall median log_2_‐transformed intensity) in the tissue of interest and below this threshold in all the other normal tissues (with three exception for PE transcripts). Second, we required at least a twofold change between the signal in the tissue of interest and those of all other tissues with three exceptions for PE transcripts. Third, statistically significant changes across the samples were identified using a LIMMA statistical test with the false discovery rate (FDR) adjustment method (*P* ≤ 0.01). Transcripts preferentially or specifically expressed in testis as compared to the other somatic normal tissues are termed PET and SET, respectively. Furthermore, two types of transcripts, which show expression signals in testis or germ cells that are in the upper quartile of the overall log_2_‐transformed expression matrix are termed specifically expressed and highly expressed in testis (SEHET) and preferentially expressed and highly expressed in testis (PEHET).

Transcripts upregulated in a given cancer subtype (UC) were identified by applying three filtration steps. First, the intensity signal had to be above the background expression cutoff (BEC = 5.5, corresponding to the overall median log_2_‐transformed intensity) in the cancer subtype of interest. Second, we required a twofold change between the signal in the cancer subtype and that of the corresponding somatic tissue. Third, the statistically significant changes across the samples were identified using a LIMMA statistical test with the FDR adjustment method (*P* ≤ 0.01). Upregulated and highly expressed in cancer (UHEC) corresponds to UC transcripts for which the expression signal in a given cancer subtype is in the upper quartile. Upregulated in cancer and not detected in healthy tissues (UCNDH) transcripts correspond to UC transcripts for which the expression signal in the corresponding somatic tissue is below the BEC. Finally, upregulated and highly expressed in cancer and not detected in healthy tissues (UHECNDH) transcripts correspond to UC transcripts for which the expression signal in a given cancer subtype is in the upper quartile and the expression signal in the corresponding somatic tissue is below the BEC.

### 2.3 Bulk and single‐cell RNA‐sequencing data processing and visualization

We integrated RNA‐sequencing data for total testis samples and enriched testicular cells (Sertoli cells, Leydig cells, peritubular cells, spermatocytes, and round spermatids) published by Ref. [13, 14] and single‐cell RNA sequencing (scRNA‐Seq) data for adult testicular cells published by Ref. [15]. The single‐cell expression profiles (scatter plots) for individual genes were generated online via the Reproductive Genomics Viewer (https://rgv.genouest.org) [16, 17].

RNA‐sequencing data for total testis, somatic tissues, and cancer samples from the Human Proteome Atlas were processed by applying pseudocounts of +1 and log_2_ transformation to the dataset (www.proteinatlas.org [18]). The signals were visualized using the *heatmap.2* package in R (CRAN); samples and genes were grouped using the default *hclust* algorithm using default color scaling for normal and testis tissues and row‐wise color scaling for cancer data provided by the TCGA consortium [19].

### 2.4 Immunohistochemistry analysis using testicular sections

Regarding human samples, the local ethics committee approved the experimental protocol “Study of Normal and Pathological Human Spermatogenesis” (registration PFS09‐015) at the French Biomedicine Agency; informed consent was obtained from all donors. The study's methodologies adhere to the standards set by the Declaration of Helsinki.

Immunohistochemical analyses using human testicular sections were carried out using a standard protocol as described [20]. Briefly, sections were deparaffinized in Ottix Plus (MM Microm Microtech, Brignais, France) and then rehydrated in ethanol and distilled water. Antigen retrieval was performed in citrate buffer pH 6.0 (Fisher Scientific, Illkirch, France) for 20 min at 95 °C. The tissues were saturated in Ultra Vision Block (Fisher Scientific) for 5 min, and antibodies against SPESP1 (1 : 5000; Sigma‐Aldrich, St. Quentin Fallavier, France; HPA 051040), GALNTL5 (1 : 500; Sigma‐Aldrich; HPA 011140), PDCL2 (1 : 2500; Sigma‐Aldrich; HPA 048260), and C11orf42 (1 : 175; Sigma‐Aldrich; HPA 063404) were applied for 16 h at 4 °C in a humidified chamber. The samples were washed and exposed to Primary Antibody Enhancer (Fisher Scientific) for 10 min and HRP polymer (Fisher Scientific) for 15 min. The substrate DAB Quanto (Fisher Scientific) was applied, and the sections were counterstained with Harris Hematoxylin (Leica Biosystems, Nanterre, France). The slides were scanned at the H2P2 platform (Biosit, Rennes, France).

### 2.5 Immunohistochemical protein detection using tissue microarrays

The IHC protocol that we used to analyze testicular sections was also applied to TMAs (Biomax, Derwood, MD, USA) that were employed to analyze SPESP1 (SK803a) GALNTL5 (EM1021, UT721, HThy‐Pap120CS‐01), PDCL2 (KD1503), and C11orf42 (TH481).

## 3 Results

### 3.1 Experimental rationale

In earlier work, we used GeneChips to determine the testicular transcriptome in biopsies from prepubertal and adult individuals, total testicular samples, and adult meiotic and postmeiotic germ cells. These analyses included an extensive set of somatic samples to classify genes into testis‐specific, preferentially expressed in testis and ubiquitous [9, 21]. We integrated our data with normal somatic controls from the NCBI's GEO repository and high‐quality GeneChip cancer expression data provided by the expO project (www.intgen.org) [22]. Our RNA profiling study is thus based on robust expression data from multiple sources that were produced using a highly standardized RNA profiling method. Our work also distinguishes itself from similar analyses by comprehensive total testis, testicular biopsy, and male germ cell sampling in combination with antibody‐based tissue profiling [23, 24, 25]. We analyzed 61 testis‐associated samples including total testis (two samples), seminiferous tubules (2), meiotic (2) and postmeiotic (2) germ cells, and pubertal (15) and adult (38) testicular biopsies. Furthermore, we processed 544 samples from 45 normal somatic tissues that we used as controls and 2281 samples corresponding to 214 cancer subtypes from 23 distinct tissue origins.

### 3.2 CT gene identification and definition of expression‐level cutoff values

**Fig. 1:**
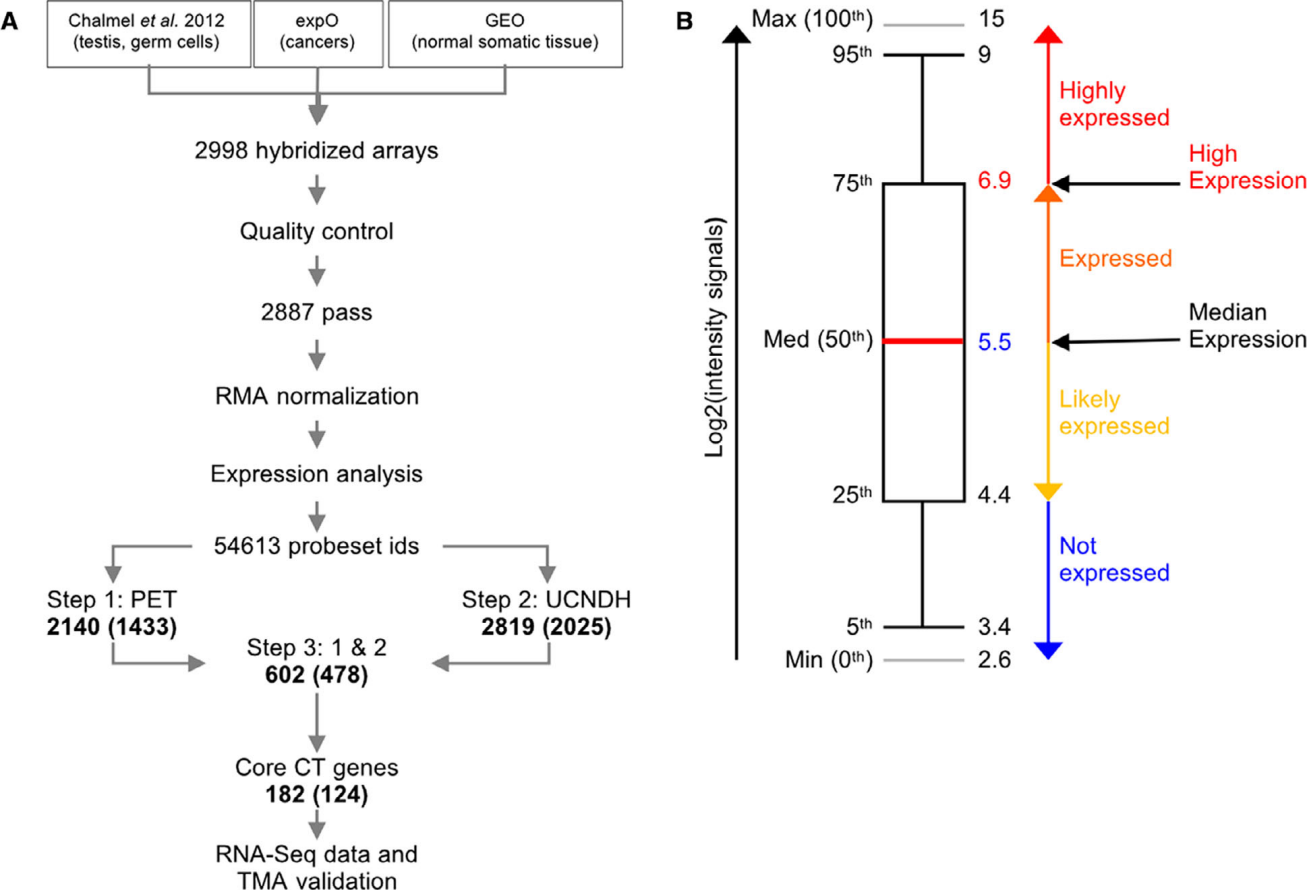
RNA expression data analysis procedure. (A) The experimental protocol and analysis procedure for CT gene screening are shown as a flowchart. Data from 2998 human U133 Plus 2.0 GeneChip were processed and normalized as shown. Probeset numbers are indicated for filtration steps, and corresponding gene numbers are shown in parentheses. (B) A box plot of log_2_ intensity values indicates the median signal (shown as a red line) and values that correspond to expression confidence levels (not expressed, low expression, expressed, and highly expressed) and the percentiles.

**Fig. 2:**
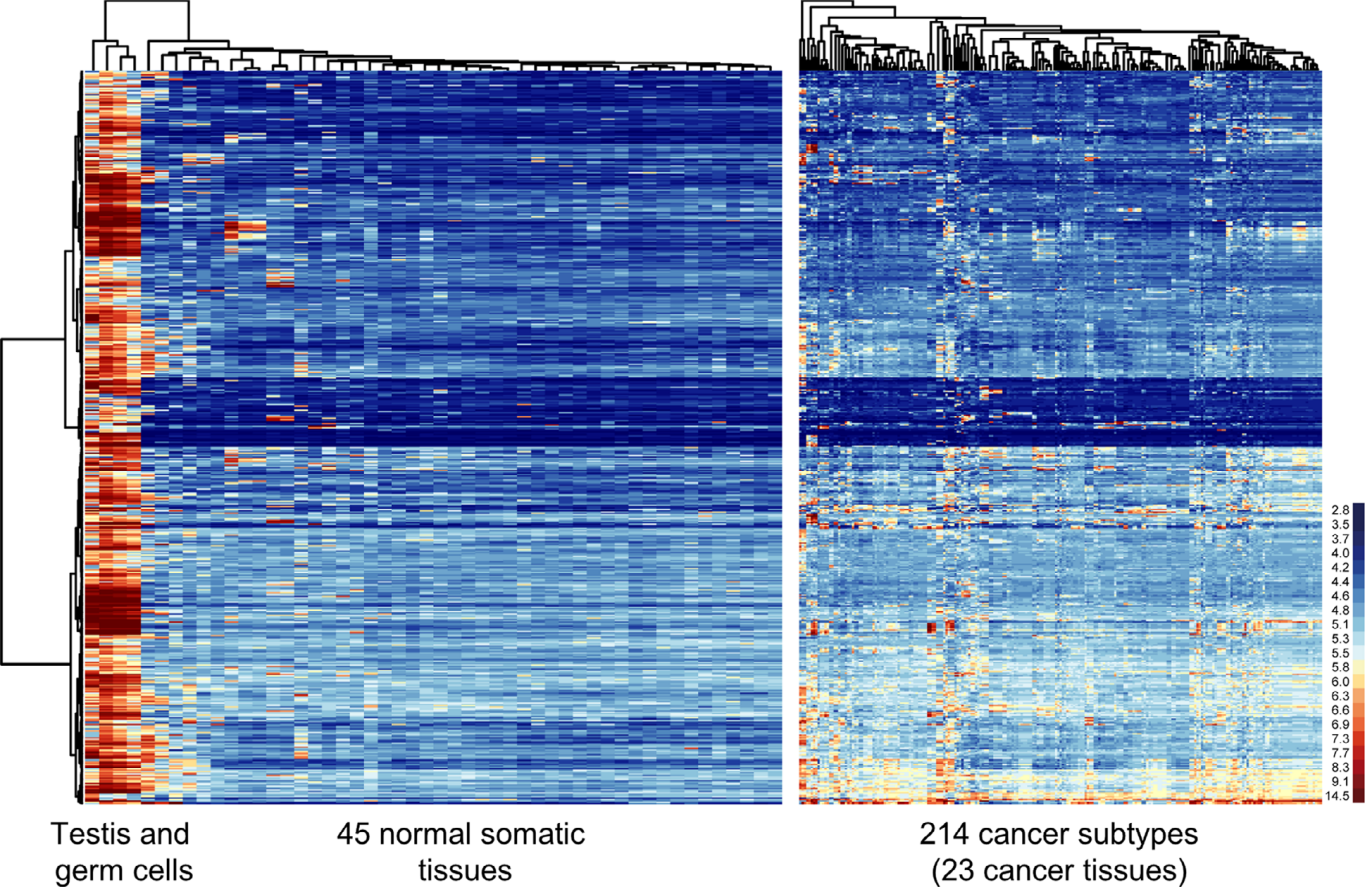
CT gene expression in testis, tumor, and normal somatic samples. False‐color heat maps are shown that display the log_2_‐transformed expression levels for testicular genes in normal somatic controls (left panel) and somatic cancers (right panel). Each line corresponds to a probeset and each column to a sample. The samples are clustered according to their expression pattern as shown by dendrograms at the top and to the left. Sample annotation categories are shown at the bottom. A color‐coded scale bar (red and blue indicate high and low expression levels, respectively) for log_2_ values is given to the right.

We assembled our testicular and germ cell data [9], cancer data from the expO project, and data for normal somatic control tissues from the GEO repository (Fig. 1A). The dataset comprises 2998 GeneChip (Human Genome U133 Plus 2.0) among which 2887 passed quality control (see methods for details; Fig. 1A). They were processed, normalized, and used in a differential gene expression analysis like in reference [9]. The median log_2_ expression value was set at 5.5, and lower and upper boundaries between the 25th and 75th percentiles defining a window of gene expression are 4.4 and 6.9, respectively. Values above the 75th percentile represent high expression and transcripts associated with values below the 25th percentile were considered to be undetectable (Fig. 1B). In step 1, among 54613 probesets we selected 2140 (corresponding to 1433 genes) that displayed significant expression in testis or germ cells, including 1285 probesets that were SE in testis. In step 2, we identified 2819 probesets (2025 genes) as being upregulated in at least one somatic cancer subtype and not expressed in the corresponding normal somatic tissue (UCNDH). The intersection of steps 1 and 2 identified 602 probe sets (478 genes) that displayed a pattern broadly corresponding to CT genes (Fig. 2; see filtering options in Supplemental File S1).

### 3.3 Extended somatic control sample sets are critical for identifying bona fide CT genes

**Fig. 3:**
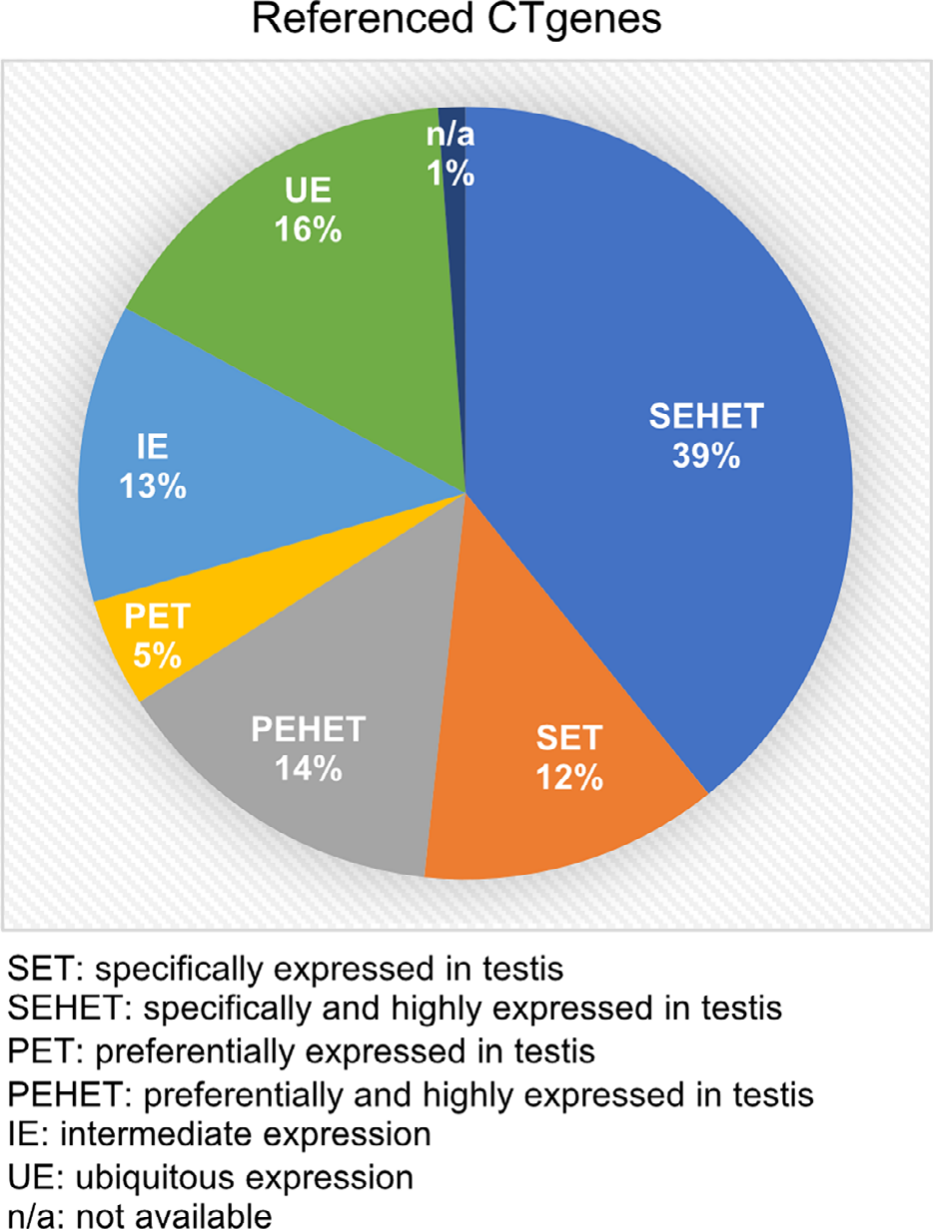
CT gene expression profiles. A pie chart shows the distribution of CT genes referenced in CT database (http://www.cta.lncc.br) among the expression classes defined by our analysis as indicated at the bottom. We employed probe sets for this analysis to avoid multiple allocations of genes to different classes. Percentages are rounded to the nearest integer.

Many putative CT genes are not testis‐specific [6]. We therefore determined expression patterns of 176 probeset‐associated mRNAs referenced in the CT database using our sample set (www.cta.lncc.br [5]). We find that 39% (69/176 probesets) are SEHET and 12% (22/176) are SET (Fig. 3; CT genes referenced in CT database are available via Supplemental File S1). However, 14% (25/176) are only PEHET and 5% (8/176) are preferentially expressed (PET), which indicates that their mRNA is detected in at least one somatic sample. For 13% (22/176), we find intermediate expression (IE, expressed in 4–10 somatic tissues) and a large group of 16% (28/176) even show ubiquitous expression (UE) in all somatic controls (Fig. 3). This pattern is unsurprising because testis contains not only germ cells but also Sertoli cells, Leydig cells, peritubular cells, smooth muscle cells, and immune cells. We note that among 1079 testicular proteins, 261 were also detected in 22 normal somatic tissues, including fallopian tube (109), cerebral cortex (46 proteins), and epididymis (28) (Fig. S1) [7]. These results underline that comprehensive control sample sets are critical for the identification of testis‐specific CT genes.

### 3.4 RNA profiling using GeneChip and RNA‐sequencing data identifies novel CT genes

To validate our filtration method, we selected 182 probesets (corresponding to 124 unique genes) that show testis‐specific expression (SET and SEHET classes), upregulation in at least one cancer subtype, and no expression in the corresponding somatic tissue (UCNDH; select SET plus SEHET and UCNDH filter options in columns G and L, respectively, in Supplemental File S1). To confirm and extend our initial GeneChip expression data, we analyzed testicular expression with our RNA‐sequencing data from total testis samples and enriched meiotic spermatocytes, postmeiotic round spermatids, and Sertoli, Leydig, and peritubular cells that were available for 115 core CT genes [13, 14]. As expected, we found that the vast majority of the core CT genes are highly induced in the male germline (see RNA‐Seq data in Fig. S2A). We then extended the analysis using single‐cell RNA‐sequencing data for testicular somatic cells (Sertoli, Leydig, and peritubular cells and macrophages), mitotic germ cells (dividing, differentiating, and differentiated spermatogonia), meiotic germ cells (leptotene, zygotene, pachytene, diplotene, and diakinesis spermatocytes), and postmeiotic germ cells (spermatids) [15]. The result confirmed that nearly all CT genes are expressed in germ cells at different stages of differentiation, including mitotic, meiotic, and postmeiotic phases of male gametogenesis (see scRNA‐Seq data in Fig. S2B).

**Fig. 4:**
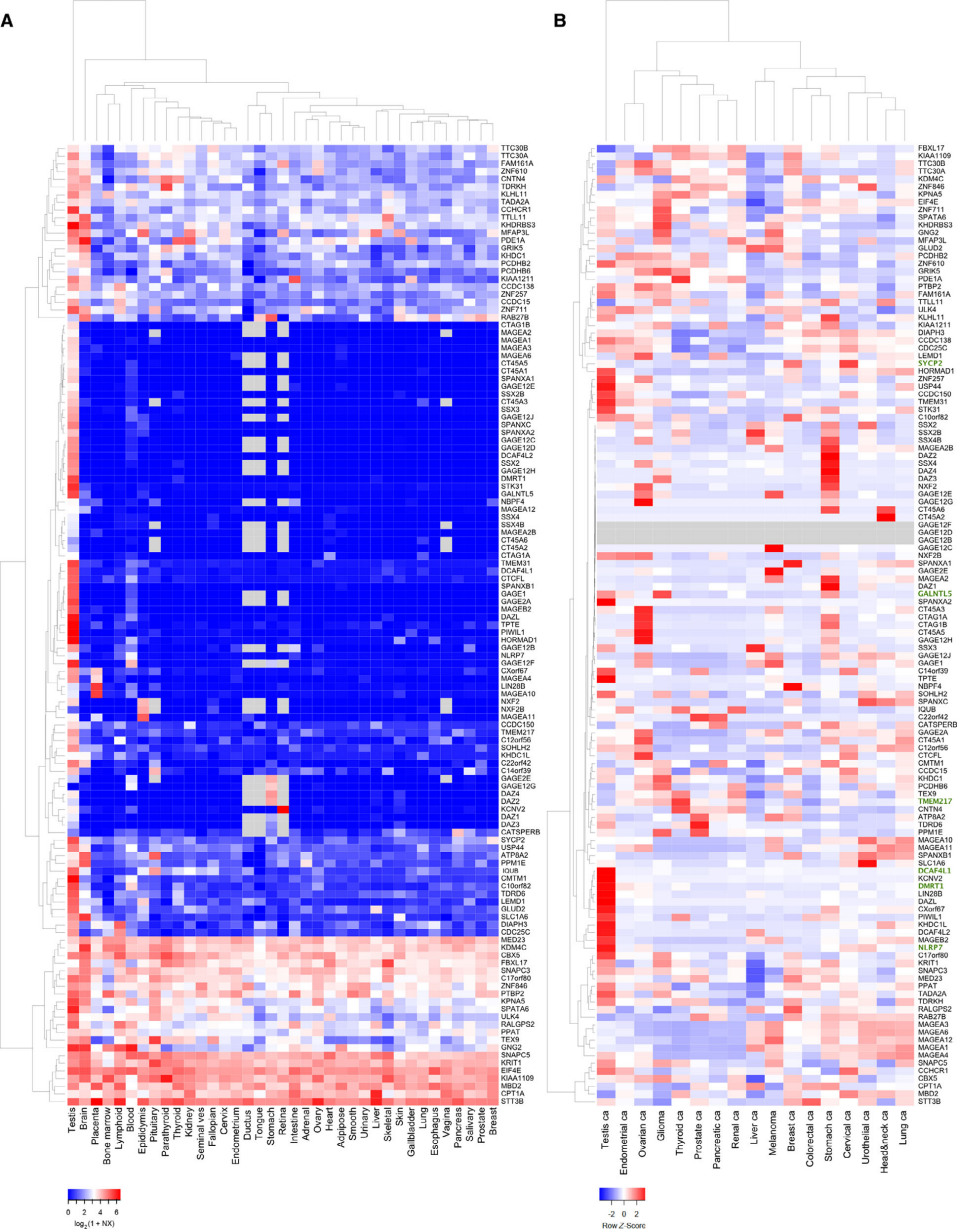
Core CT gene expression in testis, somatic tissues, and cancer. (A) A color‐coded heat map is shown for RNA‐sequencing data obtained with samples as indicated at the bottom. Each line corresponds to a gene, and each column corresponds to a sample. Gene symbols are shown to the right. Genes and samples are grouped together using the Euclidian clustering algorithm; dendrograms are shown at the top and to the left. Red and blue represent high and low expression levels. A scale is given at the bottom. (B) A color‐coded heat map is shown for RNA‐sequencing data obtained with cancer samples as indicated at the bottom. Each line corresponds to a gene, and each column corresponds to a sample. Gene symbols are shown to the right. Genes and samples are grouped together using the Euclidian clustering algorithm; dendrograms are shown at the top and to the left. Red and blue represent high and low expression levels. A scale is given at the bottom.

Next, we confirmed the testis‐specific or testis‐enriched expression pattern determined with GeneChip data for core CT genes by using RNA‐sequencing data available to us [18]. We compared expression levels in male and female gonads to 35 normal somatic tissues and found that the majority of the genes show the expected testis‐specific, preferential, or testis/brain expression patterns (Fig. 4A; see Supplemental File S2 for gene annotation and signal intensities). This result underlines that GeneChip RNA profiling data obtained with samples that were processed using highly standardized methods are reproducible in the majority of the cases, even across RNA profiling methods based on fundamentally different technologies. It is unclear why some transcripts detectable in normal somatic tissues by RNA sequencing fail to be scored as expressed by GeneChip. Different threshold levels of detection, signal processing procedures, and evolving genome annotation data that analysis procedures are based on may at least in part explain the discrepancies.

Finally, we analyzed RNA‐sequencing data from the TCGA consortium to explore the expression patterns of core CT genes in testicular and ovarian cancer versus 15 selected somatic malignancies [19, 26]. We again found the majority of them to be expressed in at least one tumor. The identification of CT genes in ovarian and testicular cancer points to meiotic functions shared by male and female gonads (Fig. 4B).

The core CT genes include CMTM1 [27], CT83 (CXorf61) [28], EZHIP (CXorf67) [29], DCAF4L2 [30], KHDRBS3 [31], LEMD1 [32], PIWIL1 [33], and SPATA6 [34], which contribute to cancer progression and metastasis. We also find HORMAD1 [35], TDRD6 [36], ZFAND4 (ANUBL1) [37], SOHLH2 [38], STK31 [39], TEX9 [40], and USP44 [41], which affect cell growth or are prognostic biomarkers. This indicates that the output of our approach is biomedically relevant.

**Fig. 5:**
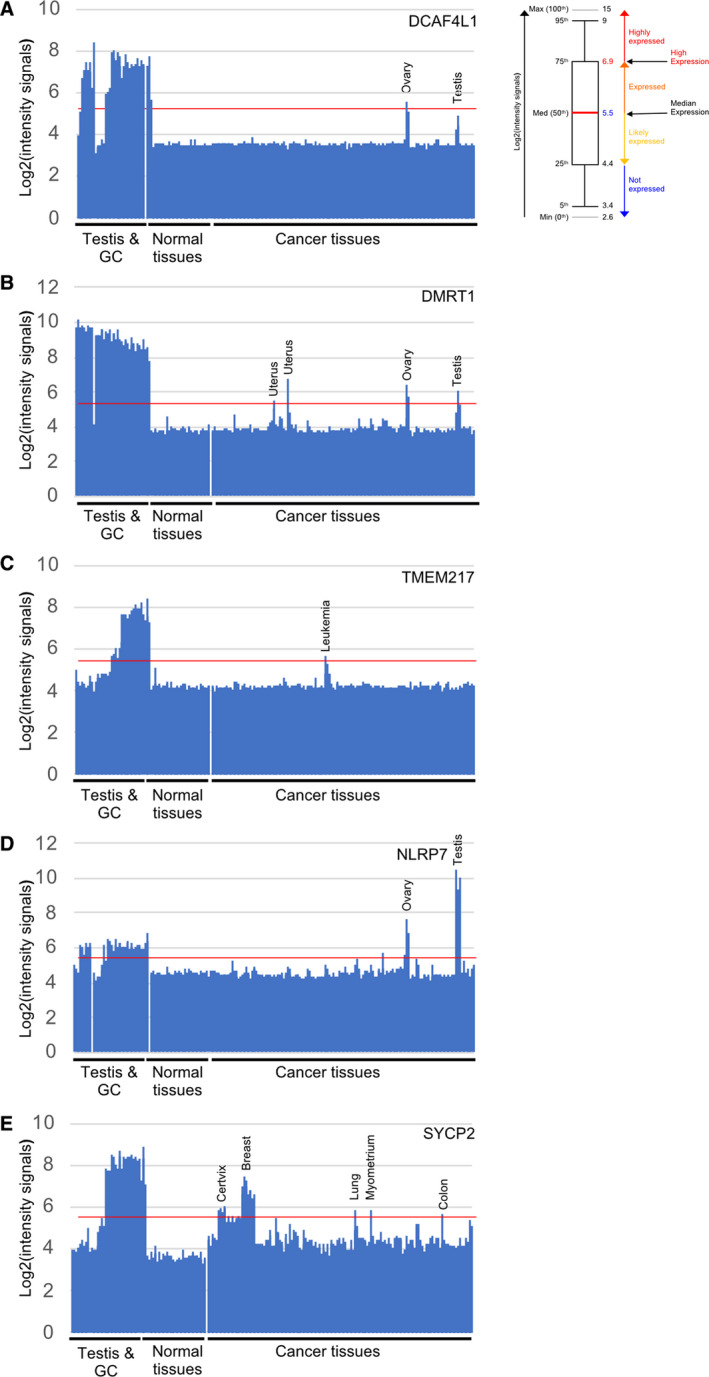
CT gene expression profiles in testis, controls, and cancer. (A‐E) Bar diagrams display the log_2_‐transformed GeneChip expression signals (*y*‐axis) obtained with total testis samples and enriched germ cells (testis and GC), controls (normal tissues), and malignant tumor samples (cancer tissues; *x*‐axis). A red line indicates the expression median. The target gene names are indicated at the top right corners. A box plot is shown like in Fig. 1B. Detailed sample annotation is available in the searchable Supplemental File␣S1.

We further investigated expression patterns of novel CT gene candidates, for which the available literature either shows testicular roles or reports critical molecular functions in somatic tumors but not both. The SEHET class gene DCAF4L1 (DDB1‐ and CUL4‐associated factor 4‐like protein 1) has no currently annotated molecular function. However, its mRNA is testis‐specific in our sample set and peaks in embryonic ovary germ cell tumor and adult seminomas (compare RNA‐sequencing data in Fig. 4 with GeneChip data in Fig. 5A). Interestingly, genetic variations in this locus were associated with hemangioblastoma, a benign brain tumor [42]. DCAF4L1 shows significant differential expression in 14 cancers versus normal controls and is detected in ovarian and testicular germ cell tumors. This includes kidney cancer and bladder cancer. Interestingly, high expression of DCAF4L1 is associated with a decreased probability for survival in kidney cancer patients and an increased probability in the case of bladder cancer (TCGA Consortium, http://timer.cistrome.org [43], Figs S3A and S4A).

Another SEHET‐type gene is DMRT1 (doublesex‐ and mab‐3‐related transcription factor 1), which belongs to a highly conserved family of DNA‐binding transcription factors important for development and sex differentiation. The mouse protein controls germ stem cell differentiation and the transition from mitotic growth to meiotic development in the germline (for review, see Ref. [44]). We find elevated levels of the human mRNA notably in endometrium, ovary, and breast cancer samples as well as testicular cancer (Figs 4 and 5B). The latter is in keeping with genetic data that associate DMRT1 with testicular germ cell tumor susceptibility [45, 46]. Immunohistochemical data from the Human Protein Atlas (HPA) confirm that pattern in the case of breast cancer (see www.proteinatlas.org [47]). DMRT1 is upregulated in nine cancers and also shows strong signals in testicular germ cell cancers in both GeneChip and RNA‐Seq datasets. This includes endometrial cancers, for which we detect a strong expression peak that corresponds to the findings reported by the TCGA consortium (Figs 5B and S3B). We note that high expression in uterine corpus endometrial carcinoma is associated with decreased survival (Fig. S4B).

Transmembrane protein 217 (TMEM217) also shows a SEHET pattern, encodes a predicted transmembrane protein, and transcriptionally responds to an antiproliferative agent [48]. The mRNA accumulates in leukemia samples in our dataset and in various somatic malignancies, including thyroid cancer, as reported by HPA (Figs 4 and 5C; www.proteinatlas.org [47]). TMEM217's function and its expression in normal and cancer tissues are currently unknown. The gene is significantly differentially expressed in 14 cancers versus controls, including kidney renal papillary cell carcinoma and thyroid carcinoma where it is induced. We note that TMEM217 is highly induced in leukemia samples assayed with GeneChips and RNA sequencing (Figs 5C and S3C) and patients diagnosed with acute myeloid leukemia (LAML) show a decreased survival rate when the gene is highly expressed (Fig. S4C).

The SET‐type NLRP7 (NACHT, LRR, and PYD domain‐containing protein 7) is involved in the inflammatory response and was associated with myometrial invasion in human endometrial cancer [49]. The mRNA peaks in different ovarian tumors and appears to be strongly induced in testicular cancer (Figs 4 and 5D). NLRP7 is differentially expressed in 14 tumors and is also induced in ovarian cancer and testicular germ cell cancers in particular, like in our RNA profiling datasets (Figs 5D and S3D).

Finally, the SEHET class gene synaptonemal complex protein 2 (SYCP2) shows a particularly striking CT gene pattern because it is derepressed in a variety of somatic tumors, especially breast and cervical cancer to an unusually high level (Figs 4 and 5E). The protein interacts with other components of the synaptonemal complex, which ensures the separation of homologous chromosomes during the first meiotic division in male germ cells [50]. Normal expression of SYCP2 is essential for male fertility [51]. Importantly, SYCP2 was recently reported to be a biomarker for luminal A/B breast cancer [52]. SYCP2 is differentially expressed in 11 cancers and shows much stronger RNA‐Seq signals in breast and cervical cancer as compared to normal controls, which is coherent with the GeneChip data (Figs 5E and S3E).

These five cases exemplify potential CT genes that are promising candidates for functional analyses since they have been broadly associated with cell growth, differentiation, and cancer.

### 3.5 CT gene analysis at the protein level by tissue microarrays

**Fig. 6:**
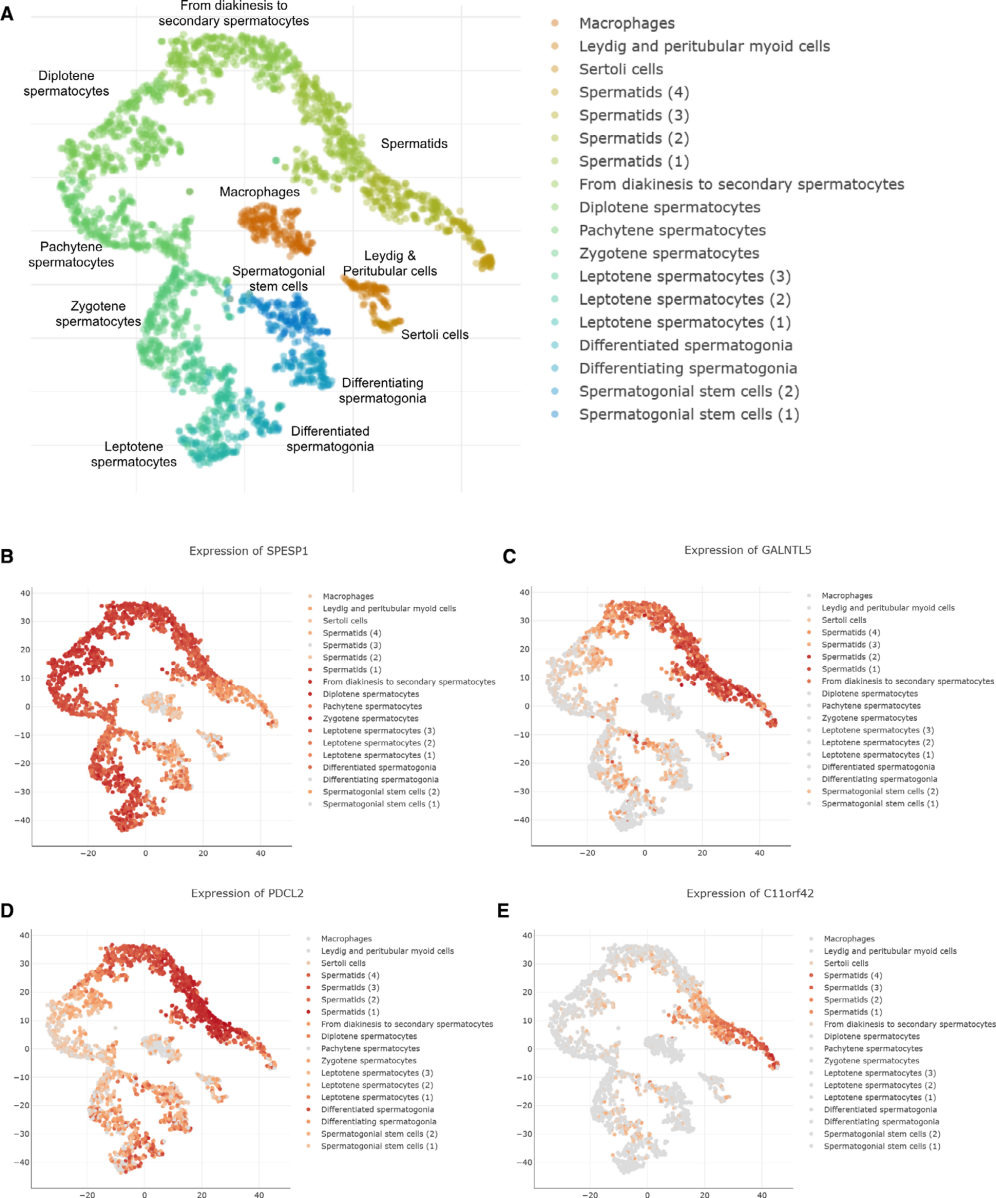
Testicular single‐cell CT gene expression profiles. (A) A schematic scatter plot of color‐coded and annotated testicular cells is shown. A legend is given to the right. Replicates are numbered. (B‐E) Scatter plots of single‐cell expression data from testicular samples are shown for four CT genes. Legends represent expression levels in cell types as indicated (shades of red and gray indicate high and low expression levels, respectively).

We next sought to further investigate the RNA/protein profiles of new CT genes for which no direct evidence was reported in the scientific literature (referenced in PubMed) that links them to altered (benign or malign) mitotic cell division [22]. To this end, we selected four candidates that showed promising mRNA/protein profiling patterns using our GeneChip expression data and IHC assays from HPA (www.proteinatlas.org [47]). We first employed published scRNA‐sequencing data to explore their expression patterns within testicular tissue [15] (Fig. 6A). The results are coherent with broad expression in the germline (SPESP1), induction in spermatocytes, and peak expression in spermatids (the core gene GALNTL5 and PDCL2) and mostly spermatid‐specific expression (C11orf42) (Fig. 6B‐E).

**Fig. 7:**
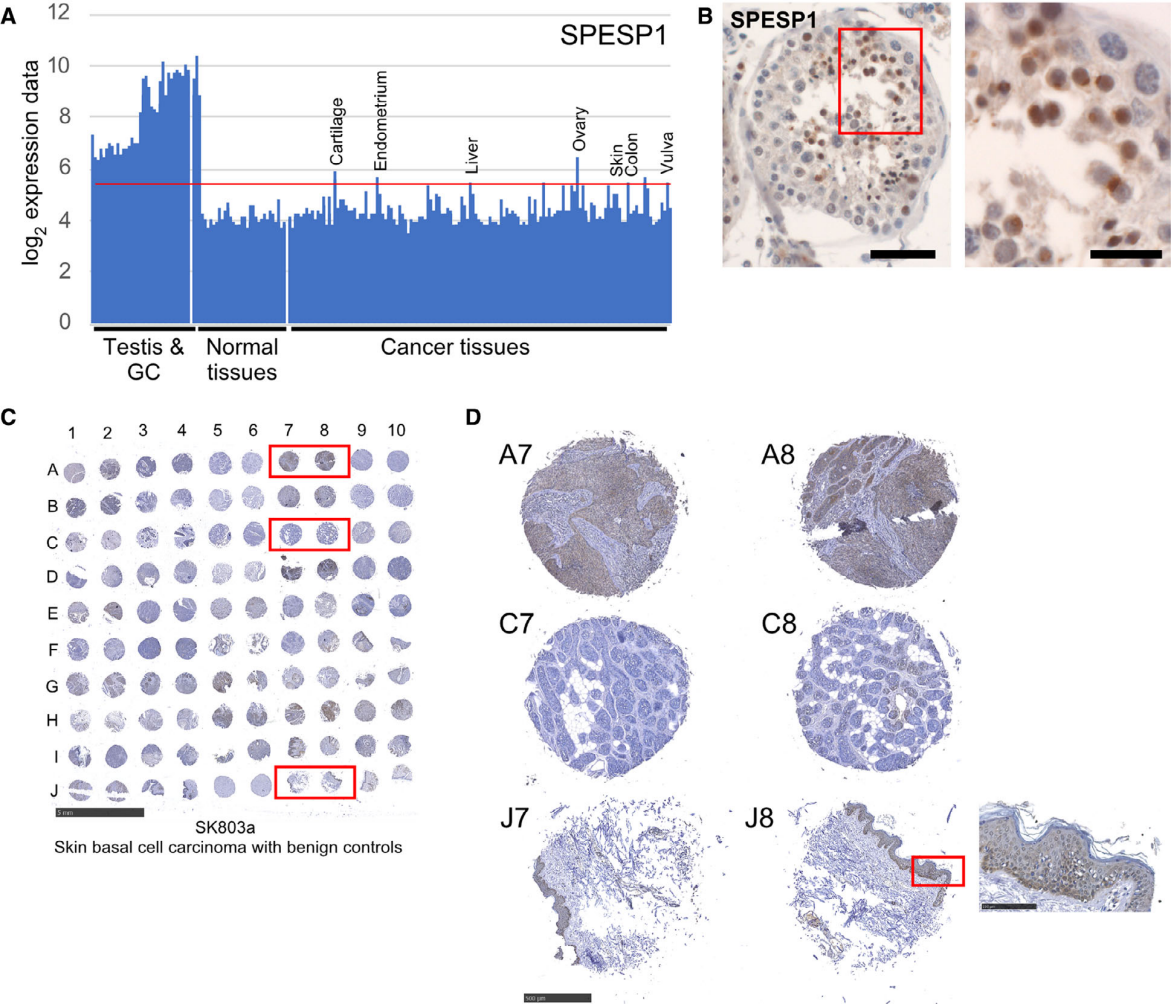
SPESP1 RNA/protein expression in skin cancer versus control samples. (A) A bar diagram is shown for the expression data like in Fig. 5. (B) Immunohistochemical staining is shown for SPESP1 on human adult testicular sections; a red rectangle marks an enlarged region given to the right. Scale bars: 50 and 20 µm. (C) An image of the hybridized TMA is shown. Samples are identified by numbers for columns and letters for rows. The TMA identifier and the cancer type are indicated at the bottom. Scale bar: 5 mm. (D) Enlarged images of typical skin cancer samples showing strong (A7‐A8) or no (C7‐C8) staining are shown at the top. Two normal skin controls (J7‐J8) are shown at the bottom. Scale bar: 500 µm. A red rectangle marks an enlarged section given to the right. Scale bar: 100 µm.

SPESP1 is an interesting gene because we classified it as specifically and highly expressed in testis (SEHET) and it encodes a membrane protein, which localizes to the sperm acrosome (see Fig. 6B for scRNA‐sequencing data and Fig. 7A for GeneChip data). Infertile male patients were found to make antibodies against SPESP1, and the mouse ortholog was shown to be involved in male fertility [53, 54, 55]. These results suggest a similarly important function in mammalian spermiogenesis and male fertility for human SPESP1. The gene is transcribed in, among several malignancies, skin, liver, and vulval cancer, which is confirmed at the protein level for skin and liver cancer by HPA (www.proteinatlas.org; Fig. 7A). We first performed an IHC assay using an HPA antibody and confirmed the protein's presence in round and elongated spermatids, which corresponds to the gene's expression in testicular samples and enriched germ cells (Fig. 7B); see also www.proteinatlas.org [47]. Next, we employed a commercial tissue microarray (TMA) covering 84 benign and malign skin tumors, 12 samples from other tumors (breast, ear, fibrous tissue, parotid gland, vulva), and four normal skin samples to analyze SPESP1's staining pattern (SK803a) (Fig. S5A). We observed that 67% (62/93) of the cancer samples on a TMA showed variable immunohistochemical signals, while the remaining cases were not stained (Fig. 7C and samples A7/8 and C7/8 in panel D). Unexpectedly, we also detected cytoplasmic staining of keratinocytes in four normal skin samples (Fig. 7D samples J7/8). The faint signal is also present in epidermal cells of normal skin samples published by HPA that were analyzed with the antibody we employed (HPA051040); however, a second antibody (HPA045936) does not yield a signal. This is in contrast with the acrosomal staining pattern that is similar for both antibodies (www.proteinatlas.org [47]). Our GeneChip data and published RNA‐sequencing data using skin samples do not indicate expression values for SPESP1 that are above background (www.proteinatlas.org [47]). Moreover, a mouse model lacking *Spesp1* shows male infertility but no defect in any somatic tissues, including skin, which argues in favor of testis‐specific roles (hence expression) [54]. It is therefore unclear whether weak signals in keratinocytes annotated as normal are true and physiologically relevant. Taken together, the data support the notion that SPESP1 is a testicular protein that accumulates in a substantial fraction of skin cancer samples.

**Fig. 8:**
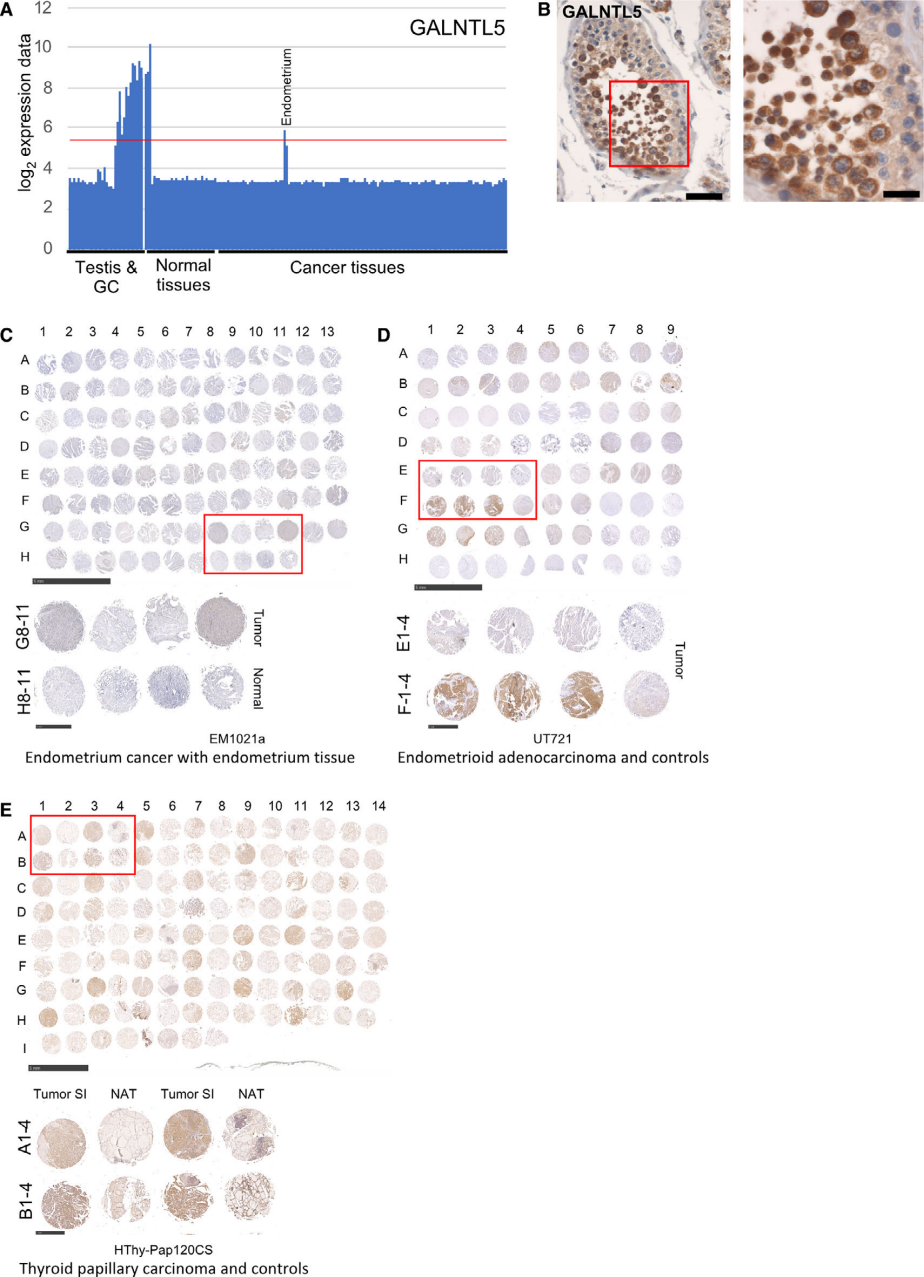
GALNTL5 RNA/protein expression in endometrial and thyroid cancer versus control samples. (A‐B) A bar diagram is shown for RNA expression data, and an image of an immunohistochemical assay is given. Scale bars: 50 and 20 µm. (C‐E). Images and schematics of hybridized cancer TMAs are given like in Fig. 7. Scale bars: 5 mm for the global view and 1 mm for the detailed view.

We next analyzed GALNTL5, which encodes a membranous inactive polypeptide N‐acetylgalactosaminyltransferase‐like protein likely important for acrosome function and proteolysis in sperm [56]. A mutation in the gene was associated with abnormal spermatogenesis in human [57]. Our GeneChip data indicate strong expression of GALNTL5 in adult testis, enriched spermatocytes, and round spermatids (Figs 6C and 8A). To test the commercially available HPA antibody (HPA011140), we first assayed GALNTL5 on adult testicular sections and observed membrane and cytoplasmic staining in spermatocytes, nuclear staining in round spermatids, and cytoplasmic staining in Leydig cells (Fig. 8B). These signals broadly correspond to patterns displayed in HPA (www.proteinatlas.org [47]). GALNTL5's RNA/protein expression profile is thus consistent with a role in the male germline as suggested by earlier work [56, 57].

GeneChip profiling data for GALNTL5 show a classical CT gene pattern in an adenocarcinoma of the endometrium. HPA's data confirm expression in endometrium at the protein level and also reveal thyroid and skin cancer samples as positively stained by IHC (Fig. 8A; www.proteinatlas.org [47]). We therefore confirmed the testicular expression of GALNTL5 using sections (Fig. 8B), and then, we employed tumor TMAs for endometrium (EM1021a, UT721) and thyroid papillary carcinoma (HThy‐Pap120CS‐01) for further analysis (Fig. S5B). Consistently, we detected cytoplasmic staining of variable intensity in 23/97 (24%) endometrium cancer samples. We also detected weak staining in 1/5 (20%) controls (EM1021a, Fig. 8C cancer samples G8‐11 and normal controls H8‐11). In a similar experiment using a different TMA 33/83 (40%), malignant samples contained detectable levels of the protein, while 0/9 controls were stained (UT721 A1, Fig. 8D cancer samples E and F1‐4). We also observed that 5/8 (63%) of the normal adjacent tissue (NAT) controls showed variable levels of staining. This again suggests that histologically normal tissues can transcribe and translate CT genes and therefore possess a molecular feature that may mark them out as (pre)malignant in spite of their normal histological appearance. Finally, we detected typically strong cytoplasmic staining in 54/58 (93%) cancerous thyroid samples, while only 14/62 (23%) of the NAT samples showed almost exclusively weak signals (HThy‐Pap120CS‐01; Fig. 8E cancer samples A1/B1 and A3/B3 and normal adjacent tissue A2/B2 and A4/B4). We conclude that histological and molecular data concur in more than three quarters of the cases and it appears that a substantial number of histologically normal tissues accumulate unphysiological levels of GALNTL5.

In summary, GALNTL5 is a testicular protein that strongly accumulates in a substantial fraction of endometrium and thyroid cancer samples. We note that HPA reports weak RNA expression signals in brain samples and two out of three brain sections show GALNTL5‐positive neuronal cells (www.proteinatlas.org [47]). This pattern is reminiscent of the testis–brain‐specific gene class, although the reliability and biological relevance of nontesticular RNA/protein expression signals for GALNTL5 remain to be determined [6].

From our current study and earlier work published by others, it emerges that RNA profiling alone is a suboptimal approach to identify testis‐specific CT genes that are likely relevant for somatic cancer progression. Major caveats of transcriptomic approaches are that RNA signal intensities used to identify target genes largely depend on the technologies and data normalization methods used and that transcribed CT gene mRNAs are not necessarily translated into physiologically relevant protein levels. Moreover, most current RNA profiling data from cancer tissues yield no information about how many and which cells in the sample express the target gene. Future work using improved single‐cell RNA‐sequencing approaches will alleviate this critical issue. Large‐scale protein profiling data obtained via IHC assays of testis, tumor, and normal somatic samples might also be a promising method complementary to RNA profiling, provided that the antibodies yield specific and reproducible results.

**Fig. 9:**
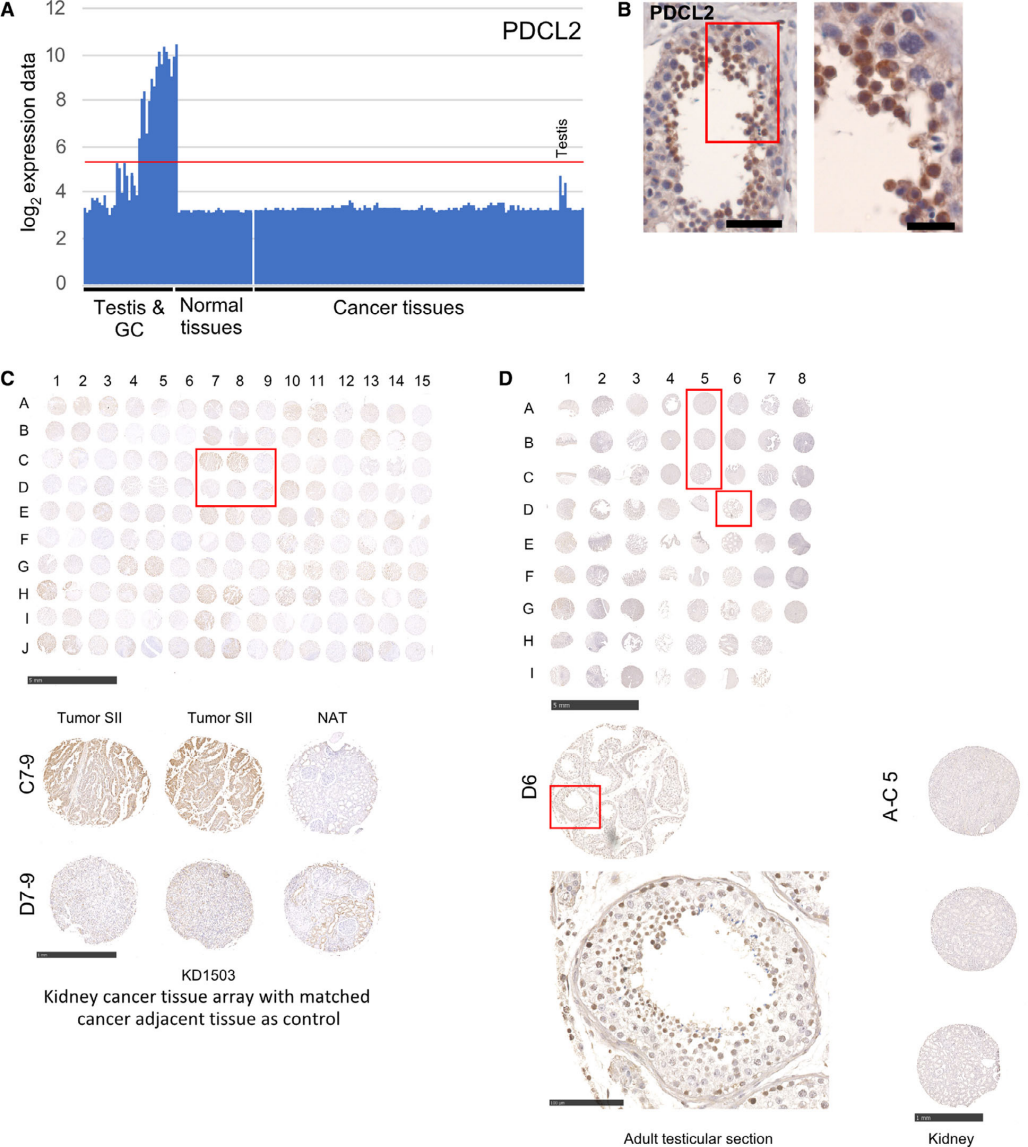
PDCL2 RNA/protein expression in kidney cancer versus control samples. (A‐B) A bar diagram is shown for RNA expression data, and an image of an immunohistochemical assay is given. Scale bars: 50 and 20 µm. (C) An image of the hybridized TMA is shown. Scale bar: 5 mm. Tumor and NAT samples are identified by numbers for columns and letters for rows. The TMA identifier and the cancer type are indicated at the bottom. Scale bar: 1 mm. (D) An image of a custom‐made TMA is given. Scale bar: 5 mm. The small rectangle over sample D6 identifies one of three testicular samples for which an enlarged images are shown at the bottom left side. Scale bar: 100 µm. The large rectangle identifies three normal kidney samples (A5‐C5); an enlarged image is shown at the bottom right side. Scale bar: 1 mm.

To further explore this protein‐based approach, we selected two testis‐specific genes for which we did not observe a typical CT gene pattern but that showed strong signals in cancer samples in HPA (www.proteinatlas.org [47]). PDCL2 encodes a phosducin‐like testis‐specific protein [58]. Human PDCL2 is induced in the male germline and expressed at the RNA and protein levels in male meiotic and postmeiotic germ cells (Fig. 6D). Furthermore, its mRNA peaks in a frequent form of testicular cancer (seminoma, Fig. 9A,B).

PDCL2 is a membrane protein that accumulates in round spermatids and in kidney cancer (www.proteinatlas.org [47]). Using an HPA antibody (HPA048260) and a TMA for renal cancer (KD1503), we found that 92/100 (92%) of malignant samples show variable levels of staining (including 16 cases for which we observed strong signals) (Fig. S5C; in Fig. 9C, compare cancer samples C7‐8/D7‐8 and normal adjacent tissues C9/D9). While 31/50 (62%) of the NAT samples were positive, only two samples displayed strong staining (Figs S5 and 9C). This demonstrates that PDCL2 protein signals for renal cancer are reproducible using custom‐made and commercial TMAs [26]. The results also reveal that a substantial proportion of NAT samples appear to accumulate low levels of PDCL2. Since healthy kidney samples displayed no PDCL2 staining on our custom‐made TMAs, while testicular germ cells are clearly marked, it is conceivable that some histologically normal tissues might already be precancerous at the molecular level (Fig. 9D testis sample D6 and kidney samples A‐C5). It is noteworthy that high expression of PDCL2 appears to be associated with decreased survival of kidney cancer patients (Fig. S6A).

**Fig. 10:**
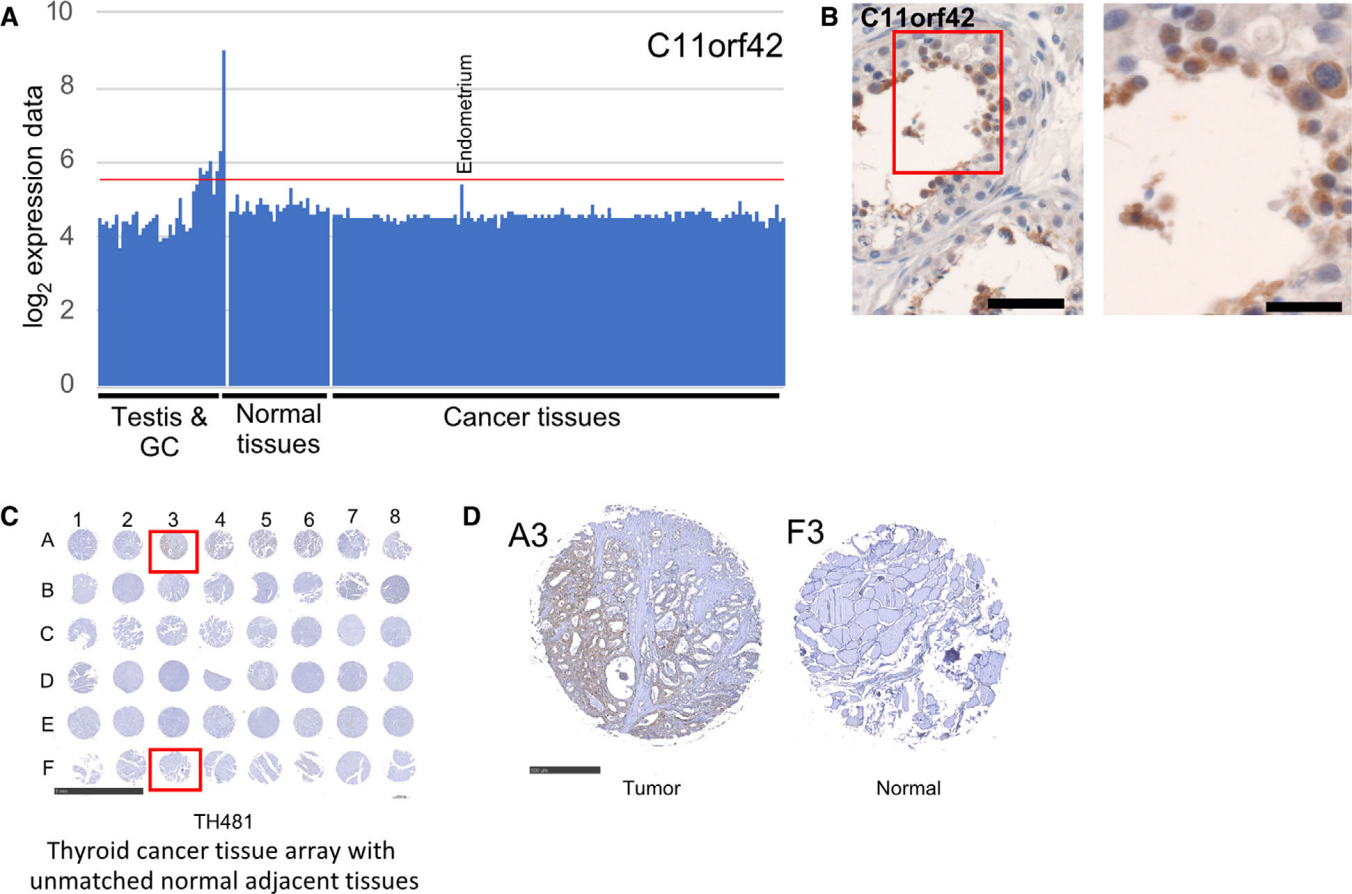
C11orf42 RNA/protein expression in thyroid cancer versus control samples. (A‐B) A bar diagram is shown for RNA expression data, and an image of an immunohistochemical assay is given. Scale bars: 50 and 20 µm. (C) An image of the hybridized TMA is shown. Samples are identified by numbers for columns and letters for rows. The TMA identifier and the cancer type are indicated at the bottom. Scale bar: 5 mm. (D) Enlarged images of a positive cancer sample (A3) and a negative control sample (F3) are given at the bottom. Scale bar: 500 µm.

C11orf42 mRNA is moderately expressed in adult testis, upregulated in enriched round spermatids and although it is likely expressed in endometrium adenocarcinoma its RNA profile in control tissues does not mark it out as a *bona fide* CT gene (Figs 6E and 10A,B). The protein is annotated as testis‐specific, and C11orf42 is detected in lung and thyroid cancer (www.proteinatlas.org [26, 47]). Consistently, we observed cytoplasmic signals for C11orf42 in 3/40 (8%) thyroid cancer samples on a commercial TMA (TH481), while 0/8 of the normal controls showed cellular staining (Figs S5D and 10C and cancer sample A3 versus normal sample F3 in panel D).

Taken together, these results underline the robustness of combined RNA/protein‐based methods to identify novel Cancer/testis genes and highlight the limits of approaches based on RNA profiling alone.

## 4 Discussion

We combined RNA/protein expression data from testis, male germ cells, normal controls, and numerous somatic cancers to identify novel CT genes suitable for biomarker discovery and mechanistic analyses in the field of molecular oncogenesis.

### 4.1 The difficulty of identifying Cancer/Testis‐specifically expressed genes

The male gonad is a complex organ and expresses the largest number of known genes among all tissues analyzed so far, together with the brain [18, 59]. However, identifying bona fide testis‐specific genes is a challenging task because somatic tissues that are used as negative controls in profiling studies are typically also composed of different cell types. When only a small subpopulation of cells in such a tissue expresses the testicular gene, its mRNAs may be diluted below the threshold level of detection, thereby yielding a false‐negative somatic control sample. This can lead to incoherent results with protein‐based assays, especially immunohistochemistry, that detects signals in any cell population (or layer) of a given somatic organ. An analysis at the single‐cell resolution level of human somatic and reproductive tissues will facilitate tackling this critical issue [15, 60, 61].

### 4.2 CT genes are promising candidates for prognostic biomarkers

CT genes represent a rich source for genes that confer oncogenic properties when abnormally expressed in somatic cancer cells [62], reviewed in Ref. [3]. A growing body of evidence links CT gene expression levels to unfavorable or favorable outcomes in the progression of a variety of somatic cancers, which underlines the clinical importance of CT genes as potential biomarkers and oncogenes [3]. Among core CT genes, we identified 31 cases for which such data revealed various prognostic outcomes, including three MAGEA family members (for more details, see www.proteinatlas.org [26]). Additional examples among the core CT genes are CMTM1 (signaling molecule) and SLC1A6 (amino acid transporter), which are unfavorable expression markers for pancreatic and urothelial cancer, while KHDRBS3 (RNA splicing) and GLUD2 (glutamate dehydrogenase) are favorable markers for kidney cancer. Interestingly, the type of prognosis appears to be dependent on the tissue that is affected, because LEMD1 (signaling molecule) is a favorable marker for ovarian cancer but an unfavorable one for pancreatic cancer (see Supplemental File S2 and Fig. S7 for the complete list of genes). Such dual‐function genes have been found to be involved in cell cycle regulation; for review, see Ref. [63].

### 4.3 Novel CT genes may act as oncogenes or tumor suppressors

SPESP1 was associated with homologous recombination repair [64] (referenced in www.genomernai.org [65]) and binds LYN, a tyrosine protein kinase important for cell proliferation and the response to DNA damage [66] (http://thebiogrid.org [67] and www.nextprot.org [68]). Mouse SPESP1 interacts with CENPC1, a centromere‐binding protein that plays a role in mitotic chromosome segregation [68, 69], referenced in IntAct [70]. In summary, misexpression of SPESP1 may contribute to genetic instability and altered growth properties in somatic cancer cells.

GALNTL5 interacts with RHOU, a Rho‐related GTP‐binding protein implicated in cancer cell migration (reviewed in Ref. [71, 72, 73]), and TP53BP1, a protein involved in double‐strand break repair, response to DNA damage, and telomere dynamics [68, 74]; http://thebiogrid.org [67]. This points to a potential role for GALNTL5 in cancer cell division and resistance to chemotherapy based on drugs that introduce DNA breaks, such as 5‐fluorouracil and cisplatin [75].

A genome‐wide RNAi screen identified C11orf42 as being important for normal mammary epithelial cell growth *in vitro* [76] (www.genomernai.org [65]). In light of this potential role in cell division, it is noteworthy that C11orf42 physically interacts with the protein transporter SNX5 [77] (IntAct [70]), which is expressed in the male germline (www.proteinatlas.org [47]; www.germonline.org [78]; https://rgv.genouest.org [16]). Given that SNX5 is a negative prognostic marker for liver cancer and plays a role in promoting thyroid cancer progression by stabilizing growth factor receptors, C11orf42 may contribute to these pathological processes via its interaction with SNX5 [26, 79].

PDCL2 interacts with ACTRT1 and REST (RE1‐silencing transcription factor) (http://thebiogrid.org [67]). ACTRT1 is associated with sporadic basal cell carcinoma [80]. Mutations in REST predispose to the Wilms tumor (the most common form of childhood renal cancer), suggesting that the gene acts as a tumor suppressor in this pediatric cancer [81]. We note that high REST expression correlates with increased survival in kidney cancer contrary to PDCL2, which shows the opposite effect (http://timer.cistrome.org; Fig. S5A,B). This raises the intriguing possibility that PDCL2 may act as a negative regulator of a renal tumor suppressor gene via direct protein–protein interaction with REST.

## 5 Conclusions

The accumulating evidence underlines that CT gene products, which have been touted as major targets for tumor neoantigen‐based immunotherapies, are also interesting from an oncogenic perspective [82]. Further mechanistic studies of testicular proteins abnormally expressed in somatic cancer cells will help gain insight into molecular oncogenic processes. Such work may therefore facilitate efforts to optimize existing treatments or even open up novel therapeutic opportunities.

## Conflict of interest

The authors declare no conflict of interest.

## Author contributions

SJ performed experiments and analyzed data. FN analyzed and interpreted data. CL interpreted data and provided reagents. RM and BJ contributed to study design. FC analyzed data, and MP conceived the study, interpreted data, and wrote the manuscript. All authors contributed to the manuscript.

## Supporting information


**Fig.␣S1.** Detection pattern of testicular proteins in somatic tissues.

**Fig.␣S2.** Core CT gene expression in testis and male germ cells.

**Fig.␣S3.** TCGA expression data.

**Fig.␣S4.** Kaplan‐Meyer (KM) plots for CT genes.

**Fig.␣S5.** Commercial‐ and custom cancer TMA sample annotation.

**Fig.␣S6.** Kaplan‐Meyer (KM) plot for PDCL2 and REST.

**Fig.␣S7.** Gene expression/cancer prognosis matrix.

**Supplemental File S1.** Searchable annotation and expression data.

**Supplemental File S2.** Core CT gene annotation, cancer prognosis and RNA‐Sequencing data.

## Data Availability

The entire dataset was generated with Affymetrix Human Genome U133 Plus 2.0 GeneChip (Thermo Fisher). Expression data for human testis, testicular biopsies, and enriched germ cells were described in reference [9]. Expression data for normal somatic control tissues were downloaded from the NCBI's Gene Omnibus (GEO: GSE7307, GSE6565, and GSE11839) repository [10]. Cancer expression data produced by the expO project (www.intgen.org) were retrieved from GEO (GSE2109) and combined with two other datasets (GSE10802 and GSE6891).

## References

[1] Simpson AJ , Caballero OL , Jungbluth A , Chen YT & Old LJ (2005) Cancer/testis antigens, gametogenesis and cancer. Nat Rev Cancer 5, 615–625. 16034368 10.1038/nrc1669

[2] Maxfield KE , Taus PJ , Corcoran K , Wooten J , Macion J , Zhou Y , Borromeo M , Kollipara RK , Yan J , Xie Y *et␣al*. (2015) Comprehensive functional characterization of cancer‐testis antigens defines obligate participation in multiple hallmarks of cancer. Nat Commun 6, 8840. 26567849 10.1038/ncomms9840 PMC4660212

[3] Gibbs ZA & Whitehurst AW (2018) Emerging contributions of cancer/testis antigens to neoplastic behaviors. Trends Cancer 4, 701–712. 30292353 10.1016/j.trecan.2018.08.005 PMC6366644

[4] Gordeeva O (2018) Cancer‐testis antigens: unique cancer stem cell biomarkers and targets for cancer therapy. Semin Cancer Biol 53, 75–89. 30171980 10.1016/j.semcancer.2018.08.006

[5] Almeida LG , Sakabe NJ , deOliveira AR , Silva MC , Mundstein AS , Cohen T , Chen YT , Chua R , Gurung S , Gnjatic S *et␣al*. (2009) CTdatabase: a knowledge‐base of high‐throughput and curated data on cancer‐testis antigens. Nucleic Acids Res 37, D816–D819. 18838390 10.1093/nar/gkn673 PMC2686577

[6] Hofmann O , Caballero OL , Stevenson BJ , Chen YT , Cohen T , Chua R , Maher CA , Panji S , Schaefer U , Kruger A *et␣al*. (2008) Genome‐wide analysis of cancer/testis gene expression. Proc Natl Acad Sci USA 105, 20422–20427. 19088187 10.1073/pnas.0810777105 PMC2603434

[7] Djureinovic D , Fagerberg L , Hallstrom B , Danielsson A , Lindskog C , Uhlen M & Ponten F (2014) The human testis‐specific proteome defined by transcriptomics and antibody‐based profiling. Mol Hum Reprod 20, 476–488. 24598113 10.1093/molehr/gau018

[8] Djureinovic D , Hallstrom BM , Horie M , Mattsson JS , La Fleur L , Fagerberg L , Brunnstrom H , Lindskog C , Madjar K , Rahnenfuhrer J *et␣al*. (2016) Profiling cancer testis antigens in non‐small‐cell lung cancer. JCI Insight 1, e86837. 27699219 10.1172/jci.insight.86837 PMC5033889

[9] Chalmel F , Lardenois A , Evrard B , Mathieu R , Feig C , Demougin P , Gattiker A , Schulze W , Jegou B , Kirchhoff C *et␣al*. (2012) Global human tissue profiling and protein network analysis reveals distinct levels of transcriptional germline‐specificity and identifies target genes for male infertility. Hum Reprod 27, 3233–3248. 22926843 10.1093/humrep/des301

[10] Clough E & Barrett T (2016) The gene expression omnibus database. Methods Mol Biol 1418, 93–110. 27008011 10.1007/978-1-4939-3578-9_5 PMC4944384

[11] Chalmel F , Rolland AD , Niederhauser‐Wiederkehr C , Chung SS , Demougin P , Gattiker A , Moore J , Patard JJ , Wolgemuth DJ , Jegou B *et␣al*. (2007) The conserved transcriptome in human and rodent male gametogenesis. Proc Natl Acad Sci USA 104, 8346–8351. 17483452 10.1073/pnas.0701883104 PMC1864911

[12] Chalmel F & Primig M (2008) The Annotation, Mapping, Expression and Network (AMEN) suite of tools for molecular systems biology. BMC Bioinformatics 9, 86. 18254954 10.1186/1471-2105-9-86 PMC2375118

[13] Jegou B , Sankararaman S , Rolland AD , Reich D & Chalmel F (2017) Meiotic genes are enriched in regions of reduced archaic ancestry. Mol Biol Evol 34, 1974–1980. 28444387 10.1093/molbev/msx141 PMC5850719

[14] Rolland AD , Evrard B , Darde TA , Le Beguec C , Le Bras Y , Bensalah K , Lavoue S , Jost B , Primig M , Dejucq‐Rainsford N *et␣al*. (2019) RNA profiling of human testicular cells identifies syntenic lncRNAs associated with spermatogenesis. Hum Reprod 34, 1278–1290. 31247106 10.1093/humrep/dez063

[15] Wang M , Liu X , Chang G , Chen Y , An G , Yan L , Gao S , Xu Y , Cui Y , Dong J *et␣al*. (2018) Single‐cell RNA sequencing analysis reveals sequential cell fate transition during human spermatogenesis. Cell Stem Cell 23, 599–614.e594. 30174296 10.1016/j.stem.2018.08.007

[16] Darde TA , Lecluze E , Lardenois A , Stevant I , Alary N , Tuttelmann F , Collin O , Nef S , Jegou B , Rolland AD *et␣al*. (2019) The ReproGenomics Viewer: a multi‐omics and cross‐species resource compatible with single‐cell studies for the reproductive science community. Bioinformatics 35, 3133–3139. 30668675 10.1093/bioinformatics/btz047

[17] Darde TA , Sallou O , Becker E , Evrard B , Monjeaud C , Le Bras Y , Jegou B , Collin O , Rolland AD & Chalmel F (2015) The ReproGenomics Viewer: an integrative cross‐species toolbox for the reproductive science community. Nucleic Acids Res 43, W109–W116. 25883147 10.1093/nar/gkv345 PMC4489245

[18] Uhlen M , Hallstrom BM , Lindskog C , Mardinoglu A , Ponten F & Nielsen J (2016) Transcriptomics resources of human tissues and organs. Mol Syst Biol 12, 862. 27044256 10.15252/msb.20155865 PMC4848759

[19] Gao GF , Parker JS , Reynolds SM , Silva TC , Wang LB , Zhou W , Akbani R , Bailey M , Balu S , Berman BP *et␣al*. (2019) Before and after: comparison of legacy and harmonized TCGA genomic data commons' data. Cell Syst 9, 24–34.e10. 31344359 10.1016/j.cels.2019.06.006 PMC6707074

[20] Petit FG , Kervarrec C , Jamin SP , Smagulova F , Hao C , Becker E , Jegou B , Chalmel F & Primig M (2015) Combining RNA and protein profiling data with network interactions identifies genes associated with spermatogenesis in mouse and human. Biol Reprod 92, 71. 25609838 10.1095/biolreprod.114.126250

[21] Chalmel F , Lardenois A & Primig M (2007) Toward understanding the core meiotic transcriptome in mammals and its implications for somatic cancer. Ann N Y Acad Sci 1120, 1–15. 17911412 10.1196/annals.1411.010

[22] Sayers EW , Agarwala R , Bolton EE , Brister JR , Canese K , Clark K , Connor R , Fiorini N , Funk K , Hefferon T *et␣al*. (2019) Database resources of the National Center for Biotechnology Information. Nucleic Acids Res 47, D23–D28. 30395293 10.1093/nar/gky1069 PMC6323993

[23] Bruggeman JW , Koster J , Lodder P , Repping S & Hamer G (2018) Massive expression of germ cell‐specific genes is a hallmark of cancer and a potential target for novel treatment development. Oncogene 37, 5694–5700. 29907769 10.1038/s41388-018-0357-2 PMC6193945

[24] da Silva VL , Fonseca AF , Fonseca M , da Silva TE , Coelho AC , Kroll JE , de Souza JES , Stransky B , de Souza GA & de Souza SJ (2017) Genome‐wide identification of Cancer/testis genes and their association with prognosis in a pan‐cancer analysis. Oncotarget 8, 92966–92977. 29190970 10.18632/oncotarget.21715 PMC5696236

[25] Wang C , Gu Y , Zhang K , Xie K , Zhu M , Dai N , Jiang Y , Guo X , Liu M , Dai J *et␣al*. (2016) Systematic identification of genes with a cancer‐testis expression pattern in 19 cancer types. Nat Commun 7, 10499. 26813108 10.1038/ncomms10499 PMC4737856

[26] Uhlen M , Zhang C , Lee S , Sjostedt E , Fagerberg L , Bidkhori G , Benfeitas R , Arif M , Liu Z , Edfors F *et␣al*. (2017) A pathology atlas of the human cancer transcriptome. Science 357, eaan2507. 28818916 10.1126/science.aan2507

[27] Delic S , Thuy A , Schulze M , Proescholdt MA , Dietrich P , Bosserhoff AK & Riemenschneider MJ (2015) Systematic investigation of CMTM family genes suggests relevance to glioblastoma pathogenesis and CMTM1 and CMTM3 as priority targets. Genes Chromosomes Cancer 54, 433–443. 25931111 10.1002/gcc.22255

[28] Paret C , Simon P , Vormbrock K , Bender C , Kolsch A , Breitkreuz A , Yildiz O , Omokoko T , Hubich‐Rau S , Hartmann C *et␣al*. (2015) CXorf61 is a target for T cell based immunotherapy of triple‐negative breast cancer. Oncotarget 6, 25356–25367. 26327325 10.18632/oncotarget.4516 PMC4694836

[29] Jain SU , Do TJ , Lund PJ , Rashoff AQ , Diehl KL , Cieslik M , Bajic A , Juretic N , Deshmukh S , Venneti S *et␣al*. (2019) PFA ependymoma‐associated protein EZHIP inhibits PRC2 activity through a H3 K27M‐like mechanism. Nat Commun 10, 2146. 31086175 10.1038/s41467-019-09981-6 PMC6513997

[30] Wang H , Chen Y , Han J , Meng Q , Xi Q , Wu G & Zhang B (2016) DCAF4L2 promotes colorectal cancer invasion and metastasis via mediating degradation of NFkappab negative regulator PPM1B. Am J Transl Res 8, 405–418. 27158335 PMC4846892

[31] Matsumoto Y , Itou J , Sato F & Toi M (2018) SALL4 ‐ KHDRBS3 network enhances stemness by modulating CD44 splicing in basal‐like breast cancer. Cancer Med 7, 454–462. 29356399 10.1002/cam4.1296 PMC5806117

[32] Sasahira T , Kurihara M , Nakashima C , Kirita T & Kuniyasu H (2016) LEM domain containing 1 promotes oral squamous cell carcinoma invasion and endothelial transmigration. Br J Cancer 115, 52–58. 27280633 10.1038/bjc.2016.167 PMC4931378

[33] Xie K , Zhang K , Kong J , Wang C , Gu Y , Liang C , Jiang T , Qin N , Liu J , Guo X *et␣al*. (2018) Cancer‐testis gene PIWIL1 promotes cell proliferation, migration, and invasion in lung adenocarcinoma. Cancer Med 7, 157–166. 29168346 10.1002/cam4.1248 PMC5774002

[34] Huo S , Du W , Shi P , Si Y & Zhao S (2015) The role of spermatogenesis‐associated protein 6 in testicular germ cell tumors. Int J Clin Exp Pathol 8, 9119–9125. 26464655 PMC4583887

[35] Yao J , Caballero OL , Yung WK , Weinstein JN , Riggins GJ , Strausberg RL & Zhao Q (2014) Tumor subtype‐specific cancer‐testis antigens as potential biomarkers and immunotherapeutic targets for cancers. Cancer Immunol Res 2, 371–379. 24764584 10.1158/2326-6066.CIR-13-0088 PMC4007352

[36] Scanlan MJ , Welt S , Gordon CM , Chen YT , Gure AO , Stockert E , Jungbluth AA , Ritter G , Jager D , Jager E *et␣al*. (2002) Cancer‐related serological recognition of human colon cancer: identification of potential diagnostic and immunotherapeutic targets. Cancer Res 62, 4041–4047. 12124339

[37] Tang L , Chen F , Pang EJ , Zhang ZQ , Jin BW & Dong WF (2015) MicroRNA‐182 inhibits proliferation through targeting oncogenic ANUBL1 in gastric cancer. Oncol Rep 33, 1707–1716. 25682742 10.3892/or.2015.3798

[38] Ji S , Zhang W , Zhang X , Hao C , Hao A , Gao Q , Zhang H , Sun J & Hao J (2016) Sohlh2 suppresses epithelial to mesenchymal transition in breast cancer via downregulation of IL‐8. Oncotarget 7, 49411–49424. 27384482 10.18632/oncotarget.10355 PMC5226517

[39] Fok KL , Chung CM , Yi SQ , Jiang X , Sun X , Chen H , Chen YC , Kung HF , Tao Q , Diao R *et␣al*. (2012) STK31 maintains the undifferentiated state of colon cancer cells. Carcinogenesis 33, 2044–2053. 22828137 10.1093/carcin/bgs246

[40] Shuptrine CW , Ajina R , Fertig EJ , Jablonski SA , Kim Lyerly H , Hartman ZC & Weiner LM (2017) An unbiased *in␣vivo* functional genomics screening approach in mice identifies novel tumor cell‐based regulators of immune rejection. Cancer Immunol Immunother 66, 1529–1544. 28770278 10.1007/s00262-017-2047-2 PMC5854209

[41] Holland AJ & Cleveland DW (2012) The deubiquitinase USP44 is a tumor suppressor that protects against chromosome missegregation. J Clin Investig 122, 4325–4328. 23187131 10.1172/JCI66420 PMC3533566

[42] Ma D , Yang J , Wang Y , Huang X , Du G & Zhou L (2017) Whole exome sequencing identified genetic variations in Chinese hemangioblastoma patients. Am J Med Genet A 173, 2605–2613. 28742274 10.1002/ajmg.a.38350 PMC5603408

[43] Li T , Fu J , Zeng Z , Cohen D , Li J , Chen Q , Li B & Liu XS (2020) TIMER2.0 for analysis of tumor‐infiltrating immune cells. Nucleic Acids Res 48, W509–W514. 32442275 10.1093/nar/gkaa407 PMC7319575

[44] Zhang T & Zarkower D (2017) DMRT proteins and coordination of mammalian spermatogenesis. Stem Cell Res 24, 195–202. 28774758 10.1016/j.scr.2017.07.026 PMC5634931

[45] Kanetsky PA , Mitra N , Vardhanabhuti S , Vaughn DJ , Li M , Ciosek SL , Letrero R , D'Andrea K , Vaddi M , Doody DR *et␣al*. (2011) A second independent locus within DMRT1 is associated with testicular germ cell tumor susceptibility. Hum Mol Genet 20, 3109–3117. 21551455 10.1093/hmg/ddr207 PMC3131044

[46] Turnbull C , Rapley EA , Seal S , Pernet D , Renwick A , Hughes D , Ricketts M , Linger R , Nsengimana J , Deloukas P *et␣al*. (2010) Variants near DMRT1, TERT and ATF7IP are associated with testicular germ cell cancer. Nat Genet 42, 604–607. 20543847 10.1038/ng.607 PMC3773909

[47] Uhlen M , Fagerberg L , Hallstrom BM , Lindskog C , Oksvold P , Mardinoglu A , Sivertsson A , Kampf C , Sjostedt E , Asplund A *et␣al*. (2015) Proteomics. Tissue‐based map of the human proteome. Science 347, 1260419. 25613900 10.1126/science.1260419

[48] Miao Y , Cui L , Chen Z & Zhang L (2016) Gene expression profiling of DMU‐212‐induced apoptosis and anti‐angiogenesis in vascular endothelial cells. Pharm Biol 54, 660–666. 26428916 10.3109/13880209.2015.1071414

[49] Bullon P & Navarro JM (2017) Inflammasome as a key pathogenic mechanism in endometriosis. Curr Drug Targets 18, 997–1002. 27397068 10.2174/1389450117666160709013850

[50] Yang F , De La Fuente R , Leu NA , Baumann C , McLaughlin KJ & Wang PJ (2006) Mouse SYCP2 is required for synaptonemal complex assembly and chromosomal synapsis during male meiosis. J Cell Biol 173, 497–507. 16717126 10.1083/jcb.200603063 PMC2063860

[51] Schilit SLP , Menon S , Friedrich C , Kammin T , Wilch E , Hanscom C , Jiang S , Kliesch S , Talkowski ME , Tuttelmann F *et␣al*. (2020) SYCP2 translocation‐mediated dysregulation and frameshift variants cause human male infertility. Am J Hum Genet 106, 41–57. 31866047 10.1016/j.ajhg.2019.11.013 PMC7042487

[52] Wu C & Tuo Y (2019) SYCP2 expression is a novel prognostic biomarker in luminal A/B breast cancer. Future Oncol 15, 817–826. 30511892 10.2217/fon-2018-0821

[53] Fujihara Y , Murakami M , Inoue N , Satouh Y , Kaseda K , Ikawa M & Okabe M (2010) Sperm equatorial segment protein 1, SPESP1, is required for fully fertile sperm in mouse. J Cell Sci 123, 1531–1536. 20375058 10.1242/jcs.067363

[54] Ito C , Yamatoya K , Yoshida K , Fujimura L , Sugiyama H , Suganami A , Tamura Y , Hatano M , Miyado K & Toshimori K (2018) Deletion of Eqtn in mice reduces male fertility and sperm‐egg adhesion. Reproduction 156, 579–590. 30328350 10.1530/REP-18-0394

[55] Wolkowicz MJ , Digilio L , Klotz K , Shetty J , Flickinger CJ & Herr JC (2008) Equatorial segment protein (ESP) is a human alloantigen involved in sperm‐egg binding and fusion. J Androl 29, 272–282. 17978344 10.2164/jandrol.106.000604 PMC2898563

[56] Takasaki N , Tachibana K , Ogasawara S , Matsuzaki H , Hagiuda J , Ishikawa H , Mochida K , Inoue K , Ogonuki N , Ogura A *et␣al*. (2014) A heterozygous mutation of GALNTL5 affects male infertility with impairment of sperm motility. Proc Natl Acad Sci USA 111, 1120–1125. 24398516 10.1073/pnas.1310777111 PMC3903224

[57] Hagiuda J , Takasaki N , Oya M , Ishikawa H & Narimatsu H (2020) Mutation of GALNTL5 gene identified in patients diagnosed with asthenozoospermia. Hum Fertil 23, 226–233. 10.1080/14647273.2018.1562239 30628500

[58] Lou X , Bao R , Zhou CZ & Chen Y (2009) Structure of the thioredoxin‐fold domain of human phosducin‐like protein 2. Acta Crystallogr F Struct Biol Cryst Commun 65, 67–70. 10.1107/S1744309108037342 PMC2635858 19193988

[59] GTEx Consortium (2015) Human genomics. The Genotype‐Tissue Expression (GTEx) pilot analysis: multitissue gene regulation in humans. Science 348, 648–660. 25954001 10.1126/science.1262110 PMC4547484

[60] Bagnoli JW , Wange LE , Janjic A & Enard W (2019) Studying cancer heterogeneity by single‐cell RNA sequencing. Methods Mol Biol 1956, 305–319. 30779041 10.1007/978-1-4939-9151-8_14

[61] Guo J , Grow EJ , Mlcochova H , Maher GJ , Lindskog C , Nie X , Guo Y , Takei Y , Yun J , Cai L *et␣al*. (2018) The adult human testis transcriptional cell atlas. Cell Res 28, 1141–1157. 30315278 10.1038/s41422-018-0099-2 PMC6274646

[62] Maheswaran E , Pedersen CB , Ditzel HJ & Gjerstorff MF (2015) Lack of ADAM2, CALR3 and SAGE1 cancer/testis antigen expression in lung and breast cancer. PLoS One 10, e0134967. 26252478 10.1371/journal.pone.0134967 PMC4529184

[63] Lou X , Zhang J , Liu S , Xu N & Liao DJ (2014) The other side of the coin: the tumor‐suppressive aspect of oncogenes and the oncogenic aspect of tumor‐suppressive genes, such as those along the CCND‐CDK4/6‐RB axis. Cell Cycle 13, 1677–1693. 24799665 10.4161/cc.29082 PMC4111714

[64] Slabicki M , Theis M , Krastev DB , Samsonov S , Mundwiller E , Junqueira M , Paszkowski‐Rogacz M , Teyra J , Heninger AK , Poser I *et␣al*. (2010) A genome‐scale DNA repair RNAi screen identifies SPG48 as a novel gene associated with hereditary spastic paraplegia. PLoS Biol 8, e1000408. 20613862 10.1371/journal.pbio.1000408 PMC2893954

[65] Schmidt EE , Pelz O , Buhlmann S , Kerr G , Horn T & Boutros M (2013) GenomeRNAi: a database for cell‐based and *in␣vivo* RNAi phenotypes, 2013 update. Nucleic Acids Res 41, D1021–D1026. 23193271 10.1093/nar/gks1170 PMC3531141

[66] Zhu J , Larman HB , Gao G , Somwar R , Zhang Z , Laserson U , Ciccia A , Pavlova N , Church G , Zhang W *et␣al*. (2013) Protein interaction discovery using parallel analysis of translated ORFs (PLATO). Nat Biotechnol 31, 331–334. 23503679 10.1038/nbt.2539 PMC4110636

[67] Oughtred R , Stark C , Breitkreutz BJ , Rust J , Boucher L , Chang C , Kolas N , O'Donnell L , Leung G , McAdam R *et␣al*. (2019) The BioGRID interaction database: 2019 update. Nucleic Acids Res 47, D529–D541. 30476227 10.1093/nar/gky1079 PMC6324058

[68] Duek P , Gateau A , Bairoch A & Lane L (2018) Exploring the uncharacterized human proteome using neXtProt. J Proteome Res 17, 4211–4226. 30191714 10.1021/acs.jproteome.8b00537

[69] Kim J , Ishiguro K , Nambu A , Akiyoshi B , Yokobayashi S , Kagami A , Ishiguro T , Pendas AM , Takeda N , Sakakibara Y *et␣al*. (2015) Meikin is a conserved regulator of meiosis‐I‐specific kinetochore function. Nature 517, 466–471. 25533956 10.1038/nature14097

[70] Orchard S , Ammari M , Aranda B , Breuza L , Briganti L , Broackes‐Carter F , Campbell NH , Chavali G , Chen C , del‐Toro N *et␣al*. (2014) The MIntAct project–IntAct as a common curation platform for 11 molecular interaction databases. Nucleic Acids Res 42, D358–D363. 24234451 10.1093/nar/gkt1115 PMC3965093

[71] Faure S & Fort P (2015) Atypical RhoV and RhoU GTPases control development of the neural crest. Small GTPases 6, 174–177. 26555387 10.1080/21541248.2015.1025943 PMC4905279

[72] Canovas Nunes S , Manzoni M , Pizzi M , Mandato E , Carrino M , Quotti Tubi L , Zambello R , Adami F , Visentin A , Barila G *et␣al*. (2018) The small GTPase RhoU lays downstream of JAK/STAT signaling and mediates cell migration in multiple myeloma. Blood Cancer J 8, 20. 29440639 10.1038/s41408-018-0053-z PMC5811530

[73] De Piano M , Manuelli V , Zadra G , Otte J , Edqvist PD , Ponten F , Nowinski S , Niaouris A , Grigoriadis A , Loda M *et␣al*. (2020) Lipogenic signalling modulates prostate cancer cell adhesion and migration via modification of Rho GTPases. Oncogene 39, 3666–3679. 32139877 10.1038/s41388-020-1243-2 PMC7190568

[74] Woods NT , Mesquita RD , Sweet M , Carvalho MA , Li X , Liu Y , Nguyen H , Thomas CE , Iversen ES Jr , Marsillac S *et␣al*. (2012) Charting the landscape of tandem BRCT domain‐mediated protein interactions. Sci Signal 5, rs6. 22990118 10.1126/scisignal.2002255 PMC4064718

[75] Swift LH & Golsteyn RM (2014) Genotoxic anti‐cancer agents and their relationship to DNA damage, mitosis, and checkpoint adaptation in proliferating cancer cells. Int J Mol Sci 15, 3403–3431. 24573252 10.3390/ijms15033403 PMC3975345

[76] Burleigh A , McKinney S , Brimhall J , Yap D , Eirew P , Poon S , Ng V , Wan A , Prentice L , Annab L *et␣al*. (2015) A co‐culture genome‐wide RNAi screen with mammary epithelial cells reveals transmembrane signals required for growth and differentiation. Breast Cancer Res 17, 4. 25572802 10.1186/s13058-014-0510-y PMC4322558

[77] Huttlin EL , Bruckner RJ , Paulo JA , Cannon JR , Ting L , Baltier K , Colby G , Gebreab F , Gygi MP , Parzen H *et␣al*. (2017) Architecture of the human interactome defines protein communities and disease networks. Nature 545, 505–509. 28514442 10.1038/nature22366 PMC5531611

[78] Lardenois A , Gattiker A , Collin O , Chalmel F & Primig M (2010) GermOnline 4.0 is a genomics gateway for germline development, meiosis and the mitotic cell cycle. Database 2010, baq030. 21149299 10.1093/database/baq030 PMC3004465

[79] Jitsukawa S , Kamekura R , Kawata K , Ito F , Sato A , Matsumiya H , Nagaya T , Yamashita K , Kubo T , Kikuchi T *et␣al*. (2017) Loss of sorting nexin 5 stabilizes internalized growth factor receptors to promote thyroid cancer progression. J Pathol 243, 342–353. 28771744 10.1002/path.4951

[80] Bal E , Park HS , Belaid‐Choucair Z , Kayserili H , Naville M , Madrange M , Chiticariu E , Hadj‐Rabia S , Cagnard N , Kuonen F *et␣al*. (2017) Mutations in ACTRT1 and its enhancer RNA elements lead to aberrant activation of Hedgehog signaling in inherited and sporadic basal cell carcinomas. Nat Med 23, 1226–1233. 28869610 10.1038/nm.4368

[81] Mahamdallie SS , Hanks S , Karlin KL , Zachariou A , Perdeaux ER , Ruark E , Shaw CA , Renwick A , Ramsay E , Yost S *et␣al*. (2015) Mutations in the transcriptional repressor REST predispose to Wilms tumor. Nat Genet 47, 1471–1474. 26551668 10.1038/ng.3440

[82] Gjerstorff MF , Andersen MH & Ditzel HJ (2015) Oncogenic cancer/testis antigens: prime candidates for immunotherapy. Oncotarget 6, 15772–15787. 26158218 10.18632/oncotarget.4694 PMC4599236

