# Supplementary figures and images for: Combined RNA/tissue profiling identifies novel Cancer/testis genes
Source: Mol Oncol. 2021 Jun 23;15(11):3003–23. doi: 10.1002/1878-0261.12900 (PMC8564638; doi:10.1002/1878-0261.12900)

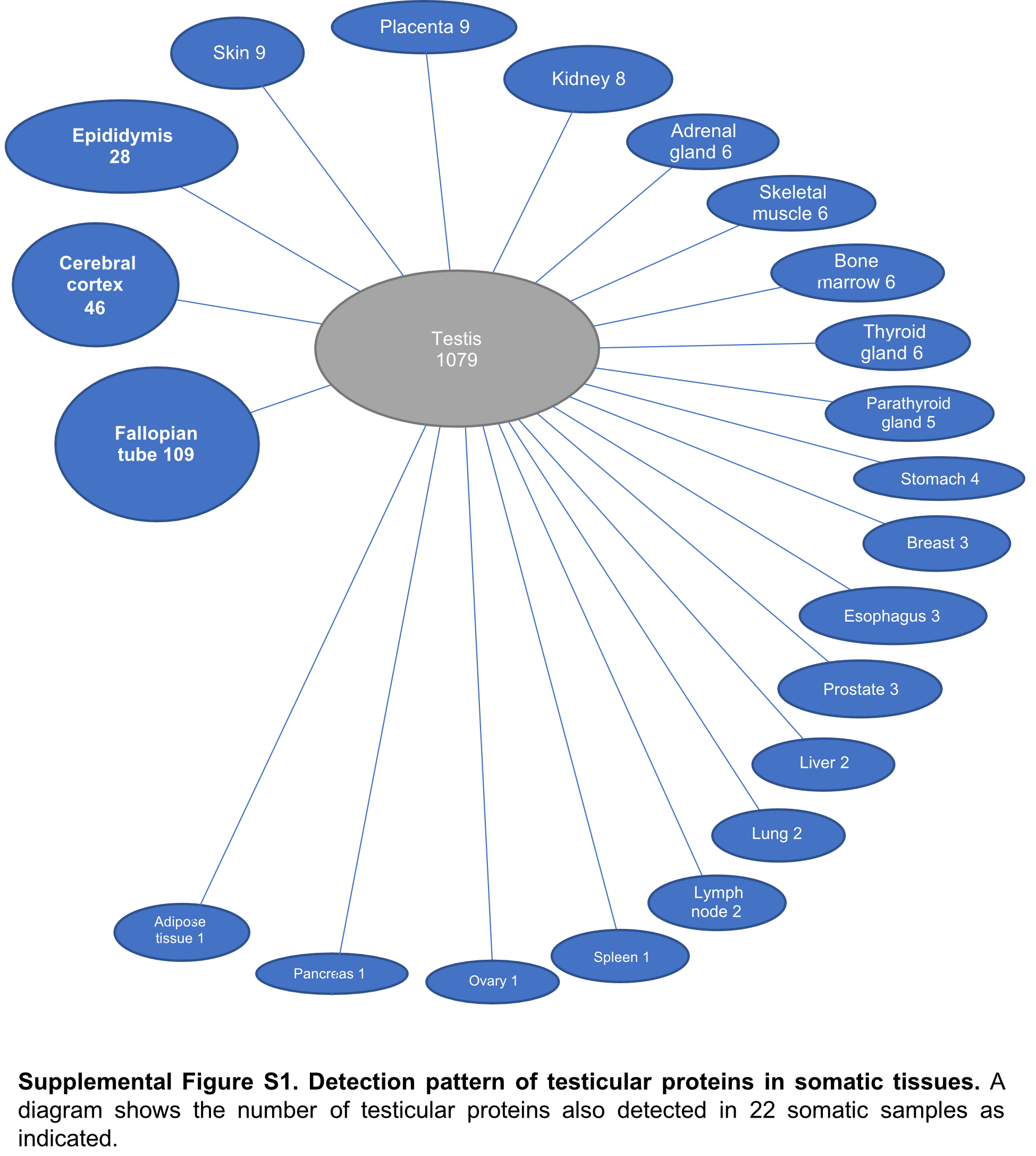

Supplement: Supplementary file 1 — Fig.␣S1. Detection pattern of testicular proteins in somatic tissues. [file MOL2-15-3003-s004.tiff]

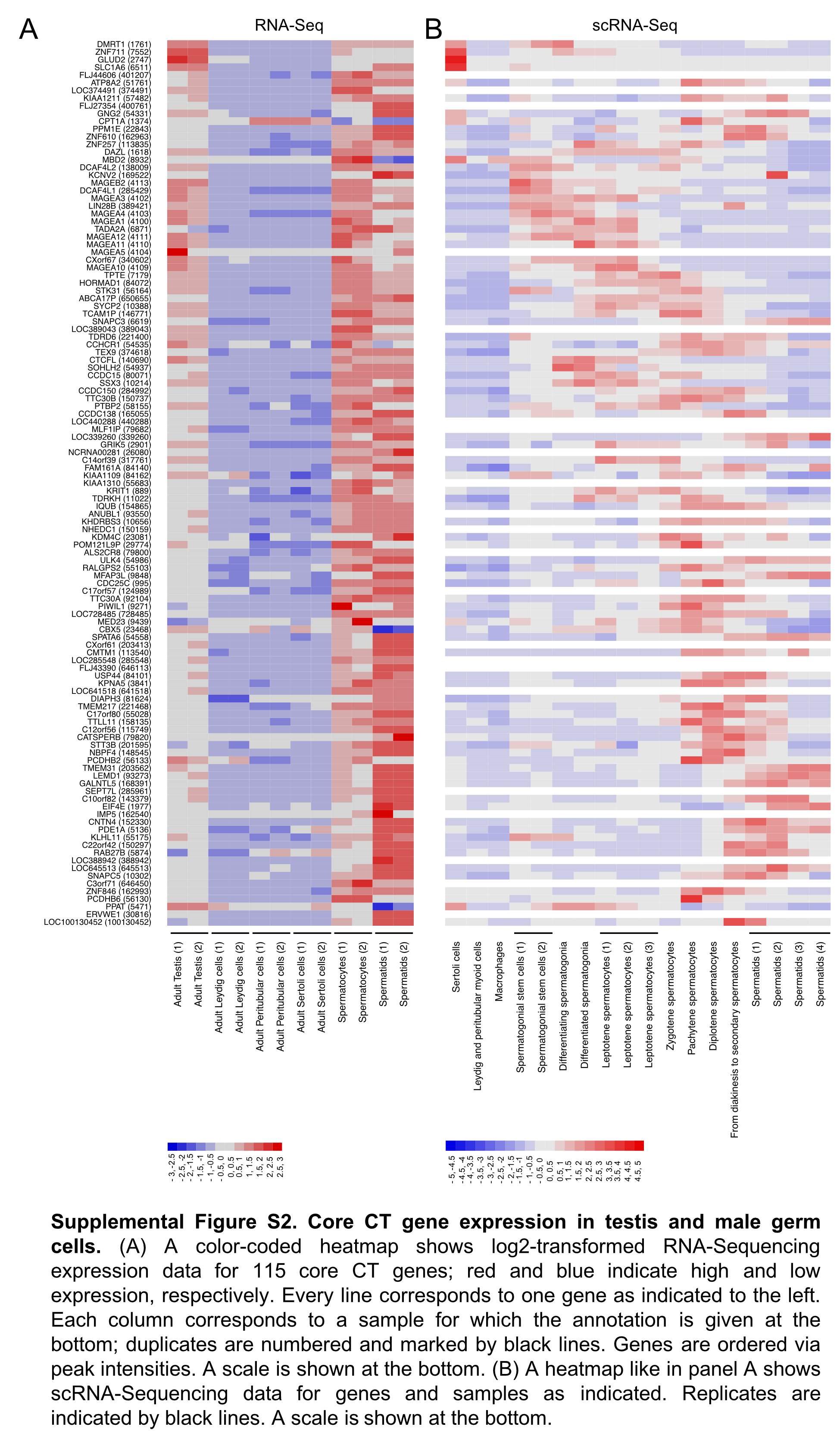

Supplement: Supplementary file 2 — Fig.␣S2. Core CT gene expression in testis and male germ cells. [file MOL2-15-3003-s001.tiff]

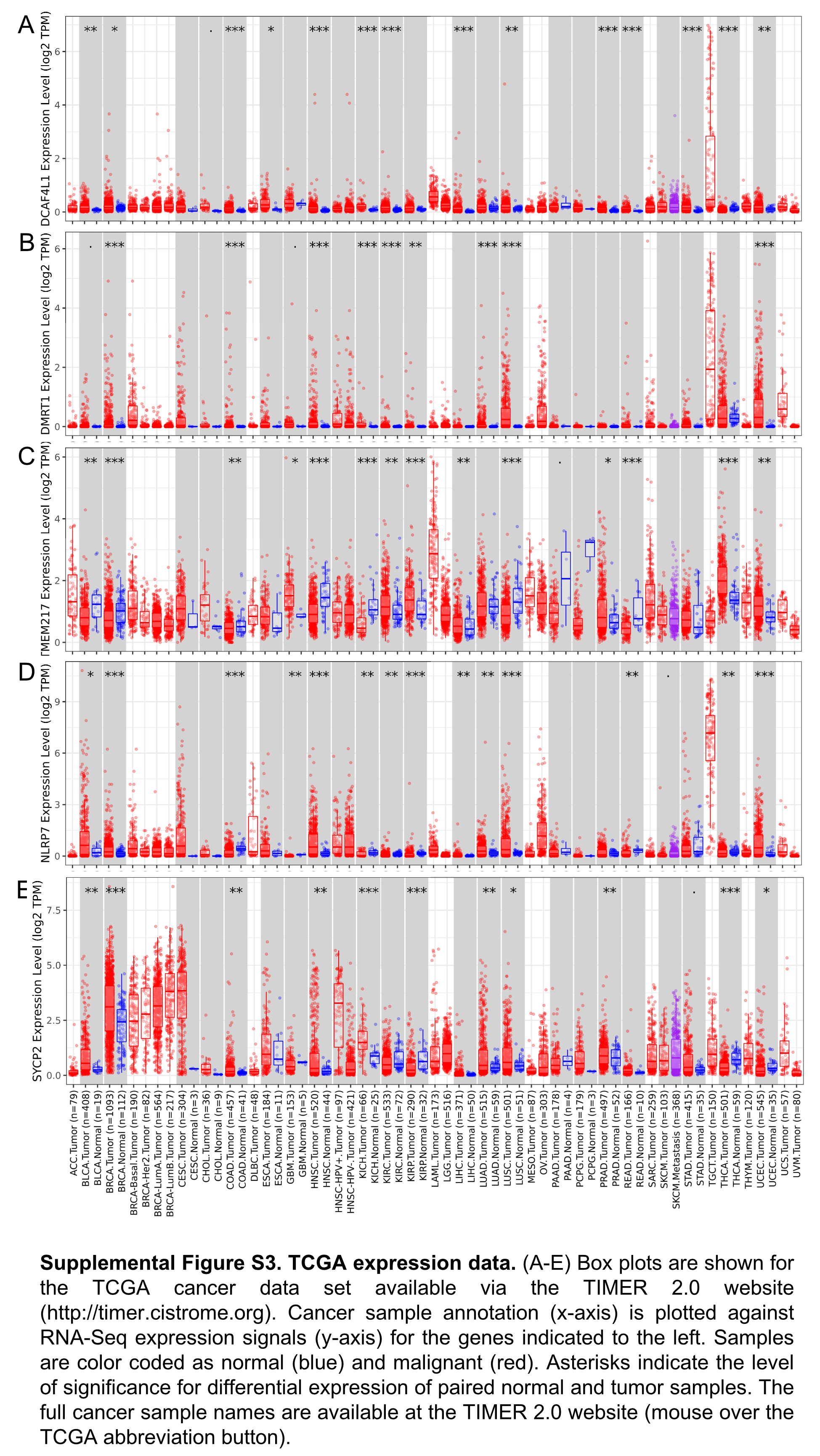

Supplement: Supplementary file 3 — Fig.␣S3. TCGA expression data. [file MOL2-15-3003-s005.tiff]

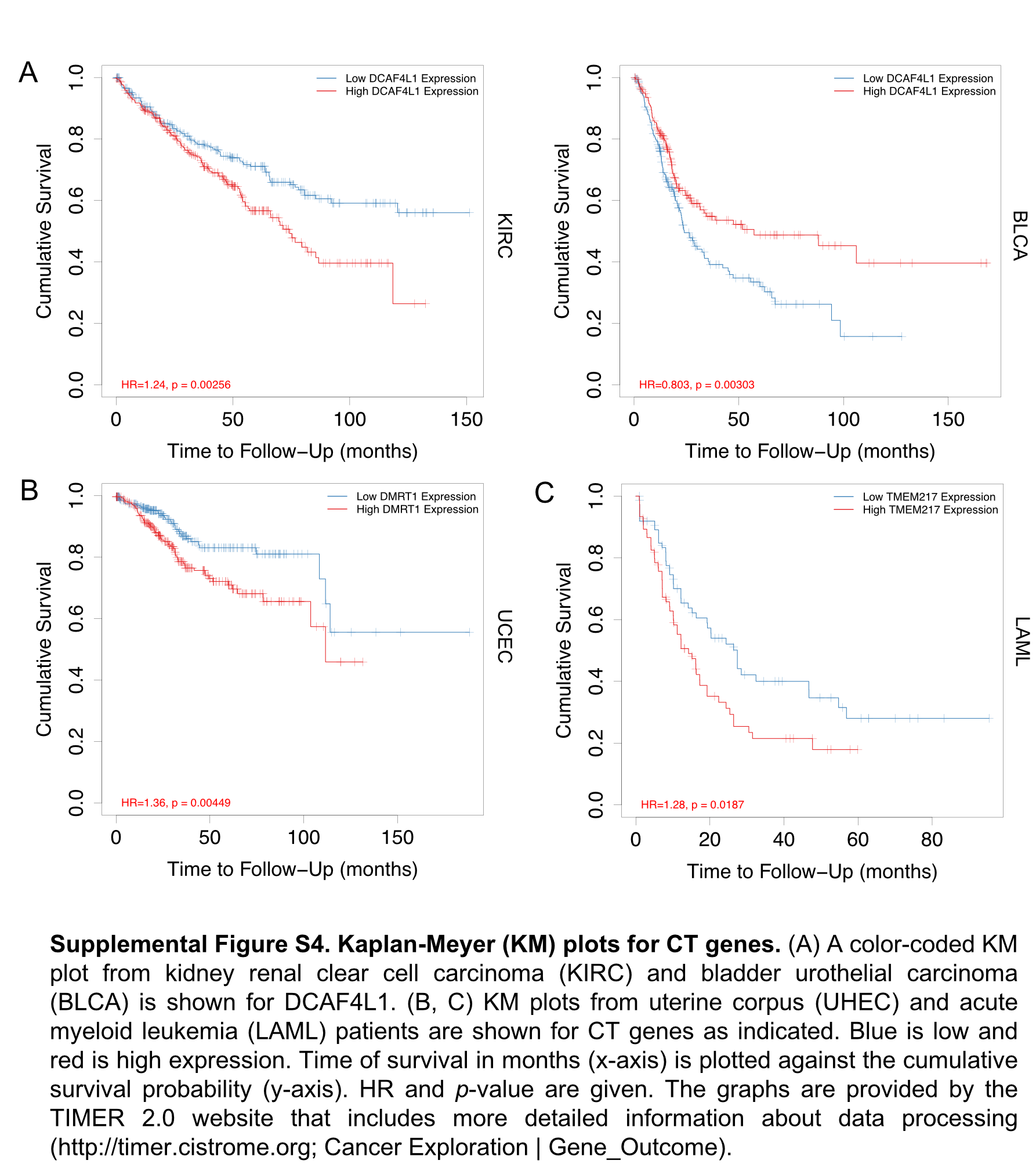

Supplement: Supplementary file 4 — Fig.␣S4. Kaplan‐Meyer (KM) plots for CT genes. [file MOL2-15-3003-s002.tiff]

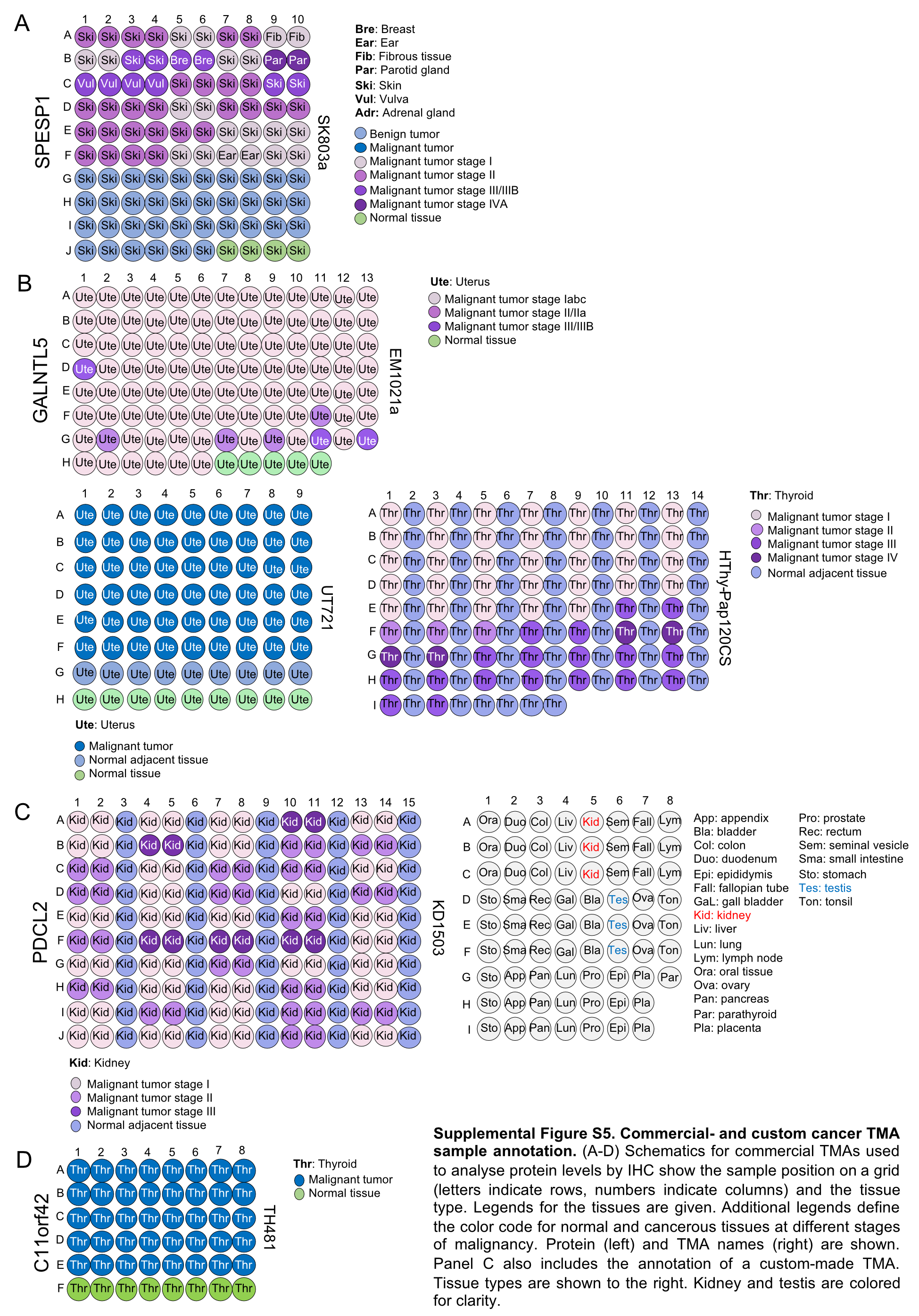

Supplement: Supplementary file 5 — Fig.␣S5. Commercial‐ and custom cancer TMA sample annotation. [file MOL2-15-3003-s008.tiff]

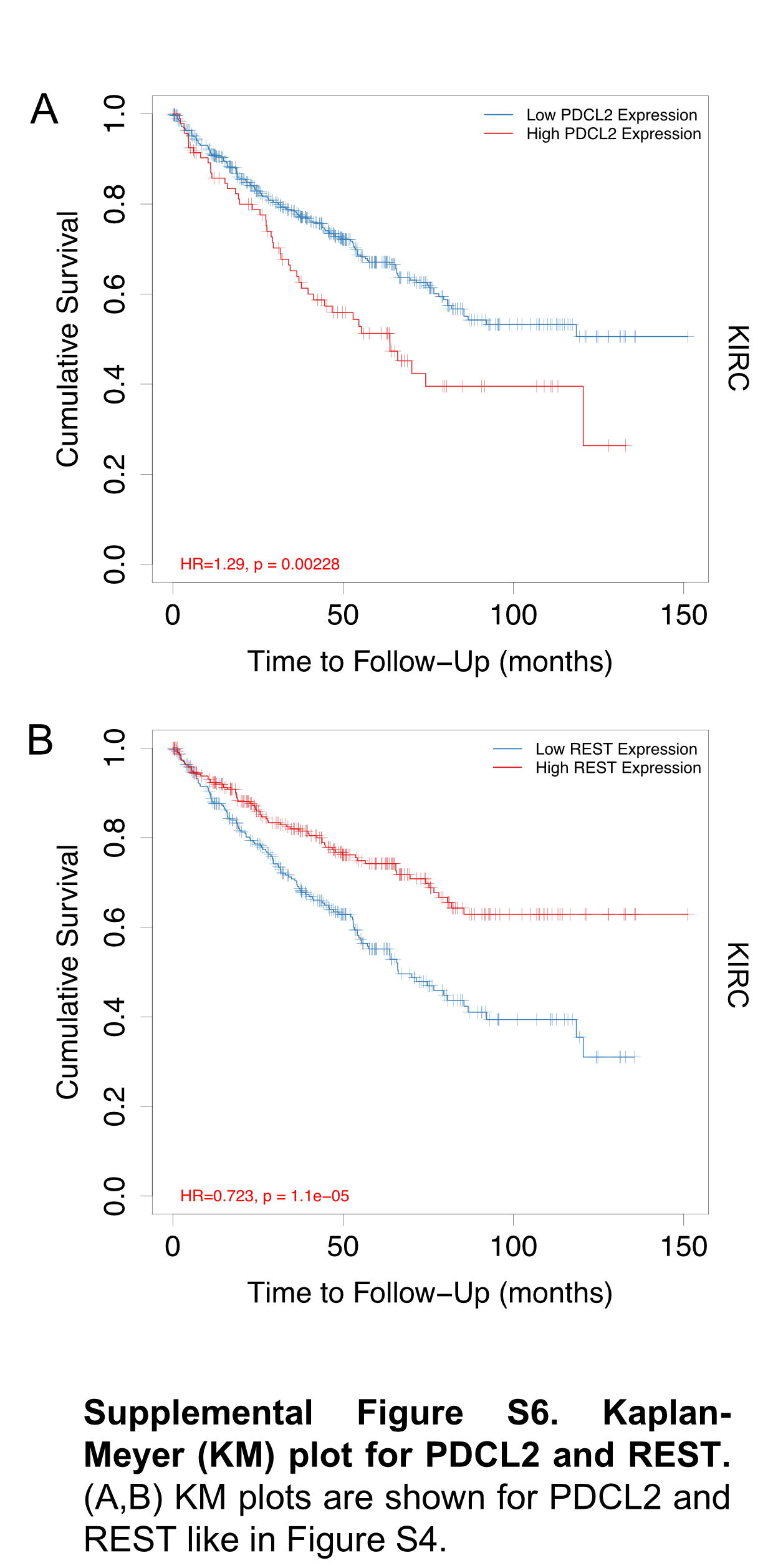

Supplement: Supplementary file 6 — Fig.␣S6. Kaplan‐Meyer (KM) plot for PDCL2 and REST. [file MOL2-15-3003-s006.tiff]

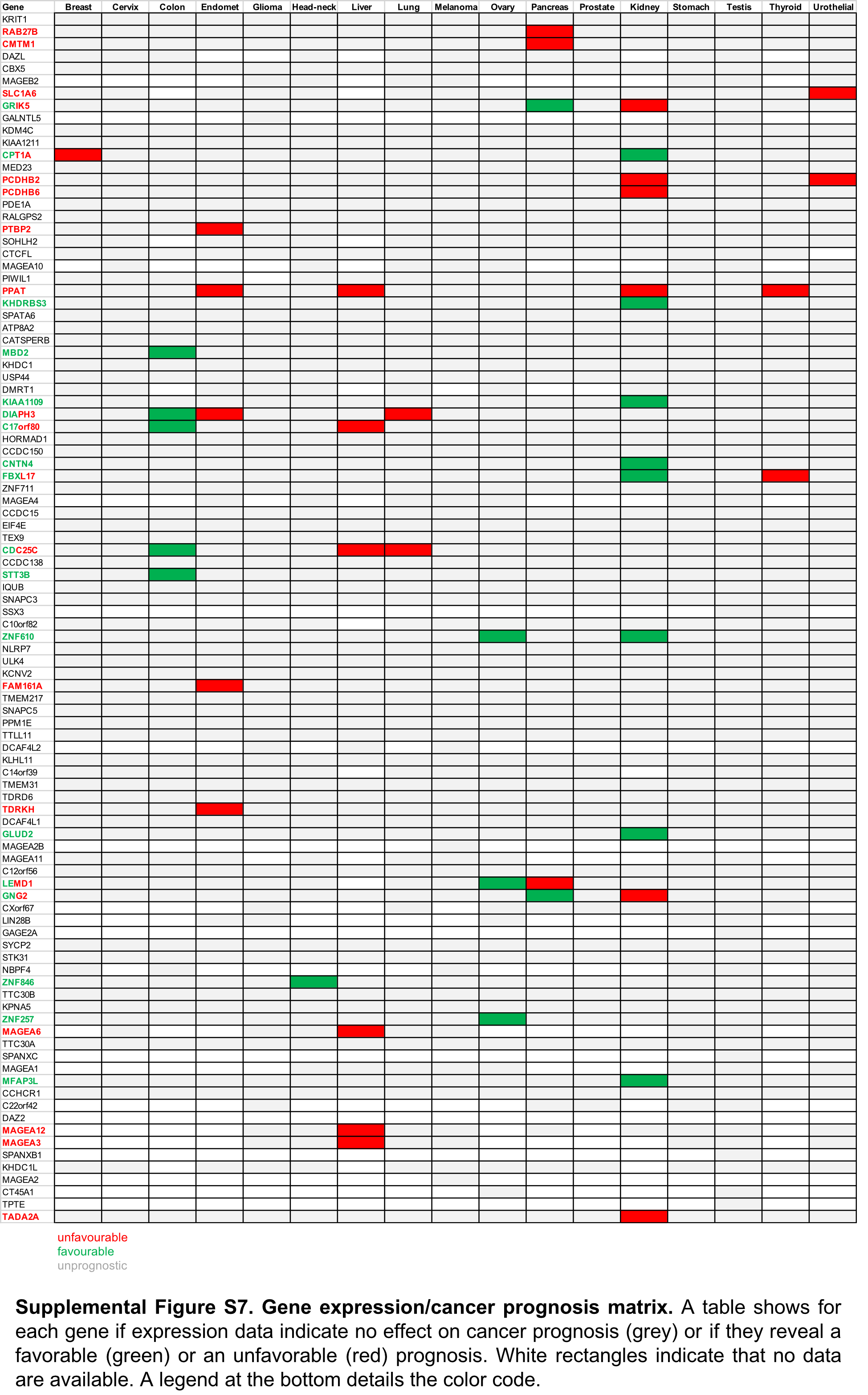

Supplement: Supplementary file 7 — Fig.␣S7. Gene expression/cancer prognosis matrix. [file MOL2-15-3003-s009.tiff]
